# Novel α-Glucosidase Inhibitory Peptides Identified In Silico from Dry-Cured Pork Loins with Probiotics through Peptidomic and Molecular Docking Analysis

**DOI:** 10.3390/nu15163539

**Published:** 2023-08-11

**Authors:** Paulina Kęska, Joanna Stadnik, Aleksandra Łupawka, Agata Michalska

**Affiliations:** Department of Animal Food Technology, Faculty of Food Science and Biotechnology, University of Life Sciences in Lublin, Skromna 8, 20-704 Lublin, Poland

**Keywords:** probiotics, bioactive peptides, dry-cured meat, fermentation

## Abstract

Diabetes mellitus is a serious metabolic disorder characterized by abnormal blood glucose levels in the body. The development of therapeutic strategies for restoring and maintaining blood glucose homeostasis is still in progress. Synthetic alpha-amylase and alpha-glucosidase inhibitors can improve blood glucose control in diabetic patients by effectively reducing the risk of postprandial hyperglycemia. Peptides of natural origin are promising compounds that can serve as alpha-glucosidase inhibitors in the treatment of type 2 diabetes. Potential alpha-glucosidase-inhibiting peptides obtained from aqueous and saline extracts from dry-cured pork loins inoculated with probiotic LAB were evaluated using in vitro and in silico methods. To identify the peptide sequences, liquid chromatography-mass spectrometry was used. For this purpose, in silico calculation methods were used, and the occurrence of bioactive fragments in the protein followed the ADMET approach. The most promising sequences were molecularly docked to test their interaction with the human alpha-glycosidase molecule (PDB ID: 5NN8). The docking studies proved that oligopeptides VATPPPPPPPK, DIPPPPM, TPPPPPPG, and TPPPPPPPK obtained by hydrolysis of proteins from ripening dry-cured pork loins showed the potential to bind to the human alpha-glucosidase molecule and may act effectively as a potential antidiabetic agent.

## 1. Introduction

Diabetes mellitus is a metabolic disorder characterized by impaired insulin secretion and/or action leading to chronic hyperglycemia, as well as alterations in carbohydrate, lipid, and protein metabolism. Diabetes, both type 1 (T1D) and type 2 (T2D), are an important problem, but are also a priority for public health agencies, the pharmaceutical and food industry, and scientists to solve, as the number of diseases and the percentage of deaths caused by diabetes increases every year. In particular, T2D accounts for more than 90% of all diabetes cases worldwide. Currently, different classes of hypoglycemic drugs are used in the treatment of type 2 diabetes, including dipeptidyl peptidase inhibitors. They act by prolonging the action of human incretin glucagon-like peptide 1 (GLP-1) and gastric inhibitory polypeptide (GIP), thereby increasing postprandial insulin secretion from pancreatic beta cells. Incretins additionally inhibit the secretion of glucagon, a hormone that increases the concentration of glucose in the blood, and inhibit the motility of the gastrointestinal tract, delaying gastric emptying. Other drugs used in pharmacology against the effects of diabetes are alpha-amylase and alpha-glucosidase inhibitors [1,2]. This class of drugs inhibits the digestion of carbohydrates by targeting these enzymes, thereby reducing postprandial hyperglycemia by delaying the hydrolysis of complex carbohydrates. The first, α-Amylase, catalyzes the initial stage of hydrolysis of polysaccharides, mainly starch, to maltose, while α-glucosidase, an enzyme associated with the small intestinal epithelium, catalyzes the hydrolysis of maltose and other disaccharides to release free glucose molecules. Thus, the strategy in which inhibitors act for these enzymes prevents glucose from being derived from complex dietary carbohydrates and released into the bloodstream [1,2]. In this way, the risk of postprandial hyperglycemia is effectively reduced.

In response to the needs of patients reporting additional, negative effects of the use of synthetic drugs, such as nausea, vomiting, or diarrhea, a new direction of research has become the search for alternatives among natural compounds that would equally effectively inhibit the action of human enzymes. A promising class of drugs that can serve as α-glucosidase inhibitors in the treatment of type 2 diabetes are bioactive compounds, such as peptides of natural origin. According to Ibrahim et al. [2], a total of 43 fully sequenced α-glucosidase inhibitory peptides have been described so far, and 13 of them had IC_50_ values several times lower than acarbose—a popular, synthetic antidiabetic drug that is an α-glucosidase inhibitor. Little is known about the bioactive compounds in food that act as a preventative or supportive factor in the treatment of diabetes when consumed in the daily diet. Although there are reports of alpha-glucosidase inhibitors in foods of plant origin, such as wheat bran and germ [3], grape pomace [4], and other plants containing bioactive ingredients, i.e., flavonoids, phenolic acids, tannins, and anthocyanins [5,6], there are only a few reports on meat as an example of food of animal origin as a source of antidiabetic ingredients. Recently, studies by Martínez-Sánchez et al. [7] assessed the cause-and-effect relationship between the consumption of dry-cured ham and cardiovascular effects, showing that consumption of dry-cured ham improves inflammatory responses and regulates thrombotic status in human clinical trials. In turn, Montoro-García et al. [8], in a similar study, showed no negative impact on the blood pressure of patients who consumed 80 g of dried ham daily. Additionally, the authors observed that total cholesterol, LDL, and basal glucose levels decreased [8], suggesting the potential of ripening meat products as a source of natural antidiabetic peptides. Previous studies have shown that raw ripened meat products are carriers of bioactive peptides with DPP-IV inhibitory activity, which may act as a strategy against type 2 diabetes mellitus [9,10,11,12]. In turn, in the study by Mora et al. [13], the α-glucosidase inhibitory potential of the peptides obtained in the water fraction of proteins extracted from traditional Spanish dry-cured ham was described for the first time. This study identified two new and active α-glucosidase inhibiting peptides that can resist digestion in the human digestive system and therefore can delay postprandial hyperglycemia in diabetic patients.

In this study, α-glucosidase-inhibiting peptides obtained from extracts (aqueous and saline) of dry-cured pork loin inoculated with LAB strains after 6 months of aging were evaluated using in vitro and in silico methods. The extracts were subjected to pepsin and pancreatin hydrolysis to obtain fragments of peptides that were potentially resistant to gastrointestinal digestion. Liquid chromatography-mass spectrometry was then used to identify the peptide sequences. Their potential for α-glucosidase inhibition was also tested in an in silico study, and pharmacokinetic properties were assessed using the ADMET approach. The most promising sequences were molecularly docked to test their interaction with the human α-glycosidase molecule (PDB ID: 5NN8).

## 2. Materials and Methods

### 2.1. Preparation of Dry-Cured Meat Products

The meat (*m. longissimus thoracis*) was cut 24 h after slaughter in a local slaughterhouse from half-carcasses of Polish large white pigs chilled to 4 °C. The next day, all loins (12) were cured with a curing mixture (20 g NaCl, 9.7 g cured salt, and 0.3 g NaNO_3_/kg loin) by surface massage. All cured batches were kept at 4 °C for 24 h to allow the curing salt to diffuse. After curing, the loin was portioned into pieces weighing about 1 kg, which were randomly divided into four experimental groups: control variant (C—not inoculated with LAB strain), LOCK (probiotic strain *Lacticaseibacillus rhamnosus* LOCK900 was used, strain deposit number: CP00548), BB12 (probiotic strain *Bifidobacterium animalis* ssp. *lactis* BB-12, strain deposit number: DSM15954), and BAUER (potentially probiotic strain *L. acidophilus* Bauer Ł0938 was used). The inoculum was applied on the surface in the amount of 0.2% (*v*/*w*) to obtain 10^6^–10^7^ CFU/g of meat, then the meat portions were suspended in a laboratory maturing chamber at a temperature of 16 ± 1 °C and relative air humidity of 75 ± 5% for 21 days, then whole loins were vacuum packed and matured at 4 ± 1 °C for 6 months (180 days).

### 2.2. Meat Protein Extraction and Hydrolysis

The water-soluble fraction (S) of meat proteins was extracted by homogenizing 10 g of meat for 5 min (T25 Basic ULTRA-TURRAX; IKA, Staufen, Germany) and distilled water (1:10 *w*/*v*) on ice and subjecting the resulting homogenate to centrifugation (10,000× *g*, 4 °C for 10 min) [14]. To prepare the salt soluble fraction (M), the precipitate resulting from the S extraction was resuspended in 0.6 M NaCl in 0.1 M phosphate buffer (pH 6.2) at a ratio of 1:6 and homogenized for 1 min on ice [15]. The resulting homogenate was kept for 18 h at 4 °C for degassing. After this time, the homogenate was subjected to centrifugation (10,000× *g*, 4 °C for 10 min), and the supernatant was filtered through Whatman No. 1 filter paper. The protein fractions obtained in this way were subjected to in vitro hydrolysis using pepsin and pancreatin [16]. In the first step, the protein extracts were adjusted to pH 2.0 with 1 M HCl and a solution of pepsin HCl (pH 2.0; 6 M) was added in an enzyme to substrate ratio of 1:100. The hydrolysis process was carried out for 120 min under the following conditions: temperature 37 °C in the dark and with continuous stirring. After the pepsin digestion step, its effect was inhibited by neutralizing the solution to pH 7.0 with 1 M NaOH. Pancreatin was then added in an enzyme to substrate ratio of 1:50 for 180 min with the conditions as before. The process of enzymatic hydrolysis was stopped by heating at 95 °C for 10 min. The hydrolysates were then dialyzed using membrane tubes (7 kDa molecular weight cutoff, Spectra/Por^®^) (Repligen Europe B.V.; Breda, The Netherlands) against phosphate-buffered saline (PBS; pH 7.4; 1:4, *v*/*v*) for 1 h at 37 °C. Obtained hydrolysates were concentrated in the evaporator and dissolved in 2 mL of 0.01 M HCl prior to chromatographic analysis.

### 2.3. Peptidomic Characteristic

#### 2.3.1. Peptide Identification by LC-MS/MS

Before the analysis, the samples were concentrated and desalted on an RP-C18 precolumn (Waters Corp., Milford, MA, USA). Separation was performed on an RP-C18 nano-Ultra Performance column (Waters, BEH130 C18 column, 75 µm i.d., 250 mm long) of a nanoACQUITY UPLC system (Warsaw, Poland) using a 180 min linear acetonitrile gradient (0–35%) at a flow rate of 250 nL/min. The column outlet was directly connected to a mass spectrometer (Orbitrap Velos, Thermo Fisher Scientific Inc., Waltham, MA, USA) for the analysis. The raw data files were preprocessed using Mascot Distiller software (version 2.4.2.0, Matrix Science Inc., Boston, MA, USA). The obtained peptide masses and their identified fragmentation pattern were compared with the protein sequence database (UniProt KB) [17] using the Mascot search engine (Mascot Daemon v. 2.4.0, Mascot Server v.2.4.1, Matrix Science, London, UK). The “mammals” option was chosen as the taxonomy constraint parameter. The search parameters applied were as follows: enzyme specificity, none; peptide mass tolerance, 5 × 10^−6^; fragment mass tolerance, 0.01 Da. The protein mass was left unrestricted, and the mass values were assumed as monoisotopic with a maximum of two missed cleavages allowed. Methylthiolation, oxidation, and carbamidomethylation were set as fixed and variable modifications. The peptide sequences from unknown original proteins were excluded. Peptide identification was performed using the Mascot search engine (Matrix Science), with a probability-based algorithm. The expected value threshold was set at 0.05 for the analysis (all peptide identification had <0.05% chance of being a random match).

#### 2.3.2. α-Glucosidase Inhibitory Activity Peptides Search

Spectrometric analysis resulted in a list of peptide sequences (a total of 8 searches: 4 for the water-soluble fraction and 4 for the salt-soluble fraction). All were tested for the presence of sequences that are potential α-glucosidase inhibitors. The search was carried out using the BIOPEP-UWM database [18]. For this purpose, in the “Calculations” tab, the frequency of occurrence of bioactive fragments in the protein sequence (parameter A) was used, which is described by the formula:

A = a/N

where: a—the number of fragments with a given activity in the protein sequence and N—the number of protein amino acid residues.

#### 2.3.3. Allergenic and ADMET Prediction

The potential effectiveness of selected peptide sequences from dry-cured pork loins was estimated by ADMET (absorption, distribution, metabolism, excretion, and toxicity) analysis. The analysis was performed using an internet platform called ADMETlab [19]. ADMET analysis included Caco-2 permeability log and human intestinal absorption (HIA) as adsorption steps, plasma protein binding (PPB), and blood–brain barrier (BBB) penetration as distribution steps, the prediction of cytochrome P450 (CYP450) 2D6 inhibition as a metabolic step, the determination of the half-life (T_1/2_) as the step of excretion, and finally the acute toxicity (LD_50_), human hepatotoxicity (H-HP), and maximum recommended daily dose (FDAMDD) was determined as the stage of toxicity. Their potential allergenicity was also tested using the AllerTOP v. 2.0 tool [20].

### 2.4. Molecular Docking

#### 2.4.1. Receptor Structure and Preparation 

**Figure 1:**
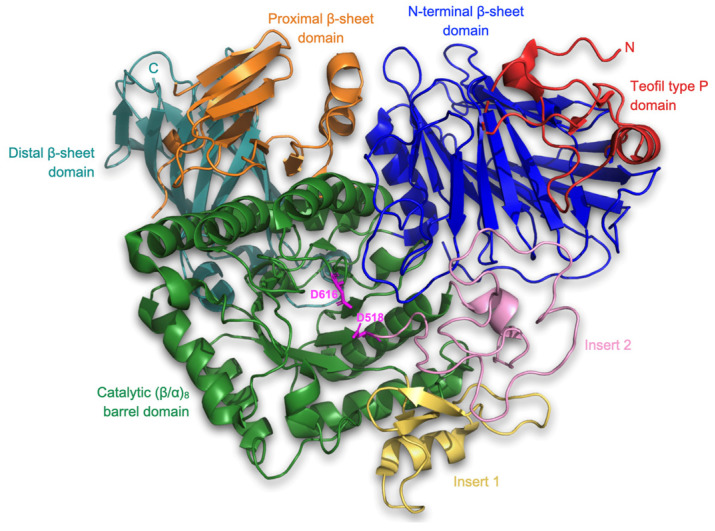
Cartoon representation of the three-dimensional structure of human lysosomal acid α-glucosidase (PDB ID: 5NN8), consisting of the N-terminal β-sheet domain (blue), trefoil type P domain (red), the proximal (orange) and distal (teal) β-sheet domains, and the catalytic (β/α)_8_ barrel domain (green) with insert I (yellow) and insert II (pink). Catalytic amino acid residues such as D616 and D518 are marked in magenta.

The receptor utilized in the molecular docking method was chain A of the crystal structure of human lysosomal acid α-glucosidase, GAA (PDB ID: 5NN8) [21,22,23]. This enzyme is essential for the degradation of glycogen within lysosomes. It exhibits the highest activity on α-1,4-glycosidic bonds, but is also capable of hydrolyzing glucans linked by α-1,6 bonds. This transmembrane protein, which is composed of 872 amino acid residues, was obtained via X-ray diffraction. The first step was to remove from the structure all entities that were not a protein receptor, and which could interfere with the course of molecular docking, including water molecules, S-hydroxycysteine, α-L-fucopyranose, 2-acetamido-2-deoxy-β-D-gluxopyranose, β-D-mannopyranose, α-D-glucopyranose, 4,6-dideoxy-4-{[{1S, 4R, 5S, 6S)-4,5,6-trihydroxy-3-(hydroxymethyl) cyclohex-2-en-1-yl]amino}-α-D-gluxopyranose, sulfate ions, chloride ions, glycerol, 1,2-ethanediol, triethylene glycol, ethylene glyxol, N-[4-hydroxymethyl-cyclohexan-6-yl-1,2,3-triol]-4,6-dideoxy-4-aminoglucopyranoside, and glycerin. The subsequent step involved preparing the receptor for molecular docking. This entailed adding hydrogen atoms and partial charges to the receptor’s structure, as well as optimizing it. To accomplish this, the AutoDockTools package, which is part of the MGLTools software (version 1.5.7) suite, was utilized [24,25]. Hydrogen atoms and partial charges (Gasteiger) were added using this package. Additionally, energy minimization was performed using General Amber Force Field (GAFF) in the Open Babel software (version 3.0.0) [26]. The file format was also appropriately converted to the one required by the QuickVina-W docking engine [27]. The prepared structure used for the study is presented in Figure 1. 

#### 2.4.2. Ligand Structures and Preparation

**Figure 2:**
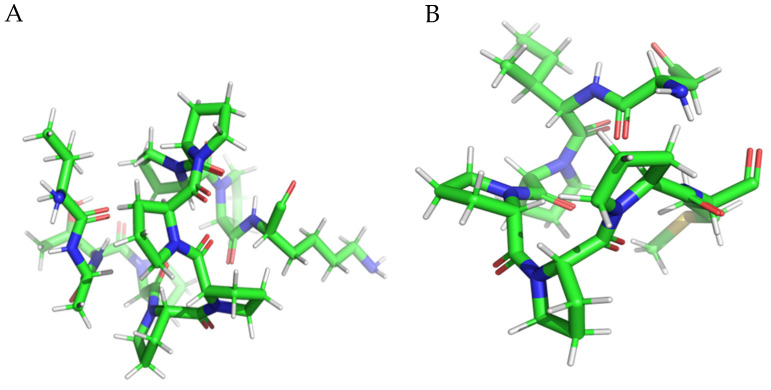

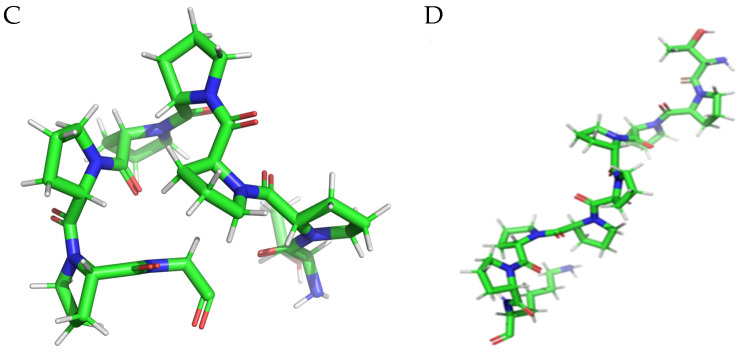
Minimized 3D structure of a peptide with the sequence VATPPPPPPPK (**A**), DIPPPPM (**B**), TPPPPPPG (**C**), and TPPPPPPPK (**D**). The color green represents C atoms, blue represents N atoms, white represents H atoms, and red represents O atoms.

The three-dimensional structures of the peptides were predicted based on their amino acid sequences using the ECEPP software (ECEPP-05 version) [28], using an Electrostatically Driven Monte Carlo (EDMC) method for peptide structure determination. The simulation proceeds through a series of Monte Carlo steps, driven by the electrostatic interaction energy between the charged residues, to pick up different variants of the peptide conformation. Finally, the resulting conformations were ranked based on their energies, and the lowest energy conformations were selected as potential 3D structures for peptide. The generated structures of each peptide were subjected to a short optimization process using GAFF force field [26]. The subsequent step involved adding partial charges to the ligand structures. Similar to the receptor, the AutoDockTools package was employed for this task. The file format was also converted to the format required by the QuickVina-W docking engine (see Figure 2). 

#### 2.4.3. Molecular Docking Analysis

To determine the binding affinity of the defined peptides to protein 5NN8 in its selected 10 binding pockets, we utilized molecular docking. We used three different types of computational software to predict the potential binding poses on the surface of the studied peptide: fpocket [29], CAVITY [30], and open-source GHECOM software (version 1.0) [31]. Peptide docking was carried out using QuickVina-W. Docking analyses were performed for the 10 selected cavities. For each cavity, the search space was set to include all atoms belonging to the cavity with some extra margin. Each of the four considered ligands were docked separately into each cavity. All analyses were performed with the exhaustiveness parameter set to 100, while all other settings were kept at their default values.

## 3. Results and Discussion

### 3.1. Peptide Characteristics

**Figure 3:**
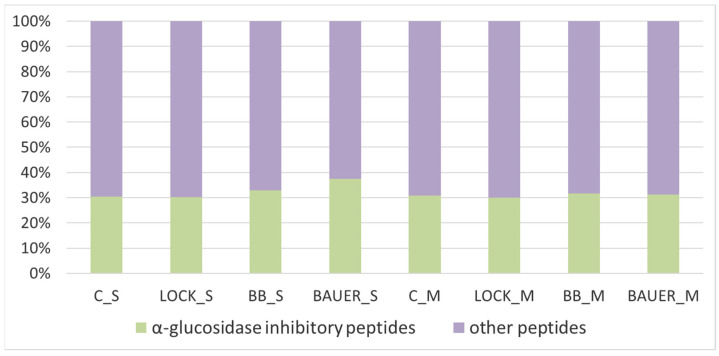
Cumulative distribution of peptides identified in test variants, including sequences potentially inhibiting α-glucosidase activity. S—peptides obtained from water-soluble fraction; M—peptides obtained from salt-soluble fraction; C, control sample; LOCK, sample inoculated with *Lacticaseibacillus rhamnosus* LOCK900; BB12, sample inoculated with *Bifidobacterium animalis* ssp. *lactis* BB-12; BAUER, sample inoculated with *Lactobacillus acidophilus* Bauer Ł0938.

In accordance with the peptidomic approach, peptides derived from variant assays (C, LOCK, BB12, BAUER, both from the S and M fraction) were analyzed by mass spectrometry, measuring their amino acid composition, molecular mass, and the type of protein from which bioinforma that are potential α-glucoside inhibitors, along with their place of occurrence, are presented in Supplement Materials (Table S1). As shown in Figure 3, both within the S fraction (extracted from the meat product with a water solvent) and the M fraction (extraction of proteins with a saline solution), a relatively equal number of sequences potentially inhibiting the activity of α-glucosidase were identified. 

Taking into account the influence of the LAB strain used, an increased number of peptide sequences with the discussed bioactivity was observed in the sample inoculated with the potentially probiotic strain *L. acidophilus* Bauer Ł0938, when the extraction of proteins from the product after 6 months of maturation was carried out with water (S).

The obtained sequences of peptides that are potential α-glucosidase inhibitors were characterized by a different value of parameter A [32]. The higher the value of parameter A, the greater part of the peptide sequence has a chance to act by interacting with the receptor present on the α-glucosidase molecule, limiting the range of its action and inhibiting the breakdown of α bonds of carbohydrates, reducing the absorption of glucose into the blood from the digestive tract, which in turn reduces glycemia after meals. Of all the sequences obtained in this analysis, only those with an A value of <0.400 were selected for further testing. Their list is presented in Table 1. The best source turned out to be the protein Phosphoglycerate mutase (B5KJG2) It is an enzyme involved in glycolysis, and its shorter fragments (peptides) can be found in the meat matrix [33]. Taking into account the method of obtaining the protein fraction, a greater number of peptide sequences with parameter A > 0.400 were associated with S than with M. Moreover, within the S fraction, the research variant subjected to spontaneous fermentation (C) was characterized by a lower number of peptides acting as potential α-glucosidase inhibitors than the variants vaccinated with the LAB starter culture. In particular, the sample inoculated with the strain *L. acidophilus* Bauer Ł0938 (BAUER_S) was characterized by almost 70% share of peptide sequences selected for further analysis. 

The ADMET profile is a useful tool for predicting the pharmacological and toxicological properties of drug candidates, especially at preclinical stages, but it has also been increasingly used for bioactive food ingredients to confirm their functional action and the role of nutrition in preventing incidence of non-communicable diseases [9,34,35,36]. Table 2 shows the pharmacokinetic properties of selected peptide sequences potentially inhibiting α-glucosidase activity by the ADMET approach.

For bioactive peptides, as well as drugs, to be effective in action they should be characterized by several features after consumption, i.e., be resistant to the action of digestive enzymes without losing biological activity, quickly and effectively absorbed (A) and distributed (D), and minimally degraded metabolically (M). There is a high probability that it will quickly reach peak blood concentration and maintain the desired level for a longer period of time before being excreted (E) [37]. Through this approach, it is possible to analyze the additional processes that peptides from food may undergo after passing through the intestinal walls into the bloodstream. Such an approach is relatively difficult to perform on humans or animals, therefore it can be performed using methods offered by in silico analysis, e.g., by using bioinformatics tools available on the ADMETlab internet platform [19]. 

The results of the allergenicity assessment (based on AllerTop v. 2.0) of these peptides were also presented, which showed a probable lack of allergenicity, with the exception of three sequences, i.e., EAPPPPAEVH, IIAPPER, and KSLRSGLLGDTLTEGGLSQLGRALREL, for which the “probable allergen” status was determined (Table 2). It should be clarified that this result does not determine their potential allergenicity (this result was not confirmed by an additional analysis carried out in the BIOPEP-UWM database), but additional analyses in this direction should be performed. In the ADMET approach the Caco-2 permeability analysis and human intestinal absorption (HIA) of peptides obtained by hydrolyzing protein extracts from dry-curing pork loin after 6 months of aging were considered as adsorption (A) steps. In this study predicted Caco-2 permeability of isolated peptides averaged −6.356, and this is lower than the optimal value according to the program criteria, i.e., the optimal logarithm of permeability should exceed −5.15, which proves the average permeability of this peptides. The exception was three peptides, i.e., SFDIPPPPMD, DLFPPPP, and IPPPPMDEK, for which the value of Caco-2 permeability was −3.223, −3.185, and −3.292, respectively. Other parameters such as human intestinal adsorption (HIA) have been used to describe parameters of feasibility of intestinal absorption, where a higher HIA means that the compound may be more efficiently absorbed by the intestine after oral administration. All analyzed sequences have a positive HIA value, on average 0.245 (Table 2). This result is lower than that reported by other food peptide researchers. As an example, 17 of the 20 analyzed sequences obtained by in silico hydrolysis of proteins from Chickpea had an HIA value > 0.3 [34]. Also, Borawska-Dziadkiewicz et al. [36] pointed out that 25 out of the 30 peptide sequences from salmon and carp revealed high predicted intestinal absorption probability with HIA > 0.3. Comparing these data, the intestinal absorption capacity of these peptides is average, and it may be a problem when we want to deliver them by food. The probable cause may be the size of the analyzed sequences. However, it should be noted that in the analysis conditions used (hydrolysis with pepsin and pancreatin) the hydrolysis of the brush border enzymes in vitro was not included, which could have affected the presented results. In addition, the examples cited were based on the in silico analysis of peptides obtained by simulating the hydrolysis of selected sequences, resulting mainly in tri-peptides and dipeptides. This approach, however, is not fully replicable in the in vitro conditions used in this study, where additional factors (e.g., intermolecular interactions in hydrolysates) may interfere with ideal hydrolysis conditions. In terms of the distribution (D) of peptides in the living organism, plasma protein binding (PPB) was also analyzed. Binding to plasma proteins may increase or decrease the bioactive effect of the drug (peptide). Therefore, the free drug concentration is a critical factor in evaluating pharmaceutical activity; the likely binding of the compounds to plasma proteins should be determined. All biopeptides are expected to be less than 90% PPB. As can be seen from the data presented in Table 2, the PPB value in this study ranged from 43.80 to 62.08%. Low molecular weight peptides can penetrate the blood–brain barrier (BBB) by slow diffusion through lipids, causing a variety of effects, including, for example, opioid side effects. The acceptable range of BBB for health promotion of candidate compounds (including drugs) is from −3.0 to 1.2 [38], which was met by all analyzed peptide fragments. The BBB coefficient value obtained in this study ranged from 0.024 (for RPPPISPPP) to 0.548 (for TPPPPPPG), which gives good safety properties of these peptides in terms of BBB penetration. The low permeability of the BBB reduces the likelihood of undesirable side effects related to the central nervous system.

In addition, all peptides were predicted to be restricted to blood (VD assumed “minus” values), which, however, does not fall within the optimal range for this parameter (i.e., 0.04–20). On the other hand, the results reported in [39] showed that acarbose, which is used as a commercial α-amylase inhibitor (same as α-glucosidase inhibitors) in the treatment of diabetes, is VD-negative. This means that the compound has some problems with intestinal absorption, however, it is effective and commercially used as a pharmacological agent. Also, peptide dipeptidyl peptidase IV inhibitors helpful in preventing the onset of diabetes derived from meat products had a negative VD determined in silico [9]. Thus, a negative VD value cannot be considered a factor that disqualifies peptides as potential antidiabetic drugs.

CYP enzymes are the main and most studied enzymes involved in various physiological and pathophysiological processes, including detoxification of xenobiotic compounds. It is estimated that only every fourth drug available on the market is not metabolized by CYP. The remaining percentage is metabolized by five major CYP isoforms, of which CYP4502D6 is involved in the metabolism of up to 75–90% of drugs [40]. In this study, metabolization (M) was assessed through potential interactions between the analyzed peptides and CYP4502D6. Cytochrome CYP24502D6 is an important enzyme in the metabolism of many xenobiotics, and therefore its inhibition may result in uncontrolled drug–drug interactions or drug lifespan. Therefore, the assessment of CYP2D6 inhibition is a key part of the discovery and development of compounds such as drugs [41]. As presented in Table 2, the analyzed peptides had both substrate and inhibitor status in relation to the CYP4502D6 enzyme. Consistent with this observation, peptide molecules have the potential to be metabolized by CYP450 enzymes. In turn, the CYP450 inhibitor status means that the molecule may hinder the biotransformation of drugs metabolized by the CYP450 enzyme. It is important that for, all analyzed peptide sequences, a stronger role as an inhibitor than a substrate was observed.

The excretion (E) capacity of the peptides was determined by determining their theoretical half-life (T_1/2_). The calculated half-life of less than 2 h was observed, which, according to the adopted criterion (>3 h), proves their low stability in the environment of the human body and high susceptibility of the peptides to degradation. However, as noted by Arámburo-Gálvez et al. [34] on the example of ACE-I inhibitors, drugs with a short serum half-life are not uncommon, although their effect can persist for hours after their consumption. The author explains that the capacity of ACE-I inhibitors to form reversible complexes with plasma proteins can serve as drug reservoirs [34,42]. It is suspected that this mechanism may also apply to other proteins acting as enzyme inhibitors, such as α-glucosidase.

The toxicity of peptides acting as potential α-glucosidase inhibitors was assessed on the basis of three independent parameters, i.e., median lethal dose (LD50), human hepatotoxicity (H-HP), and maximum recommended daily dose (FDAMDD). LD50 usually represents the acute toxicity of chemicals. It is the dose amount of a tested molecule to kill 50% of the treated animals within a given period. When comparing LD50 doses, the compound at the lower dose is more lethal than the compound at the higher LD50 dose [43]. Based on the obtained results (Table 2), the mean LD50 level was 3.060 [−log mol kg^−1^]. Taking into account the hepatotoxicity index, half of the analyzed sequences had a non-hepatotoxic status, while the other half showed a relatively low value of this index, with the highest value of 0.128 for the DIPPPPM peptide. The maximum recommended daily dose of a peptide (drug) molecule averaged 0.379 for the sequences analyzed in this study (Table 2).

### 3.2. Molecular Docking

**Figure 4:**
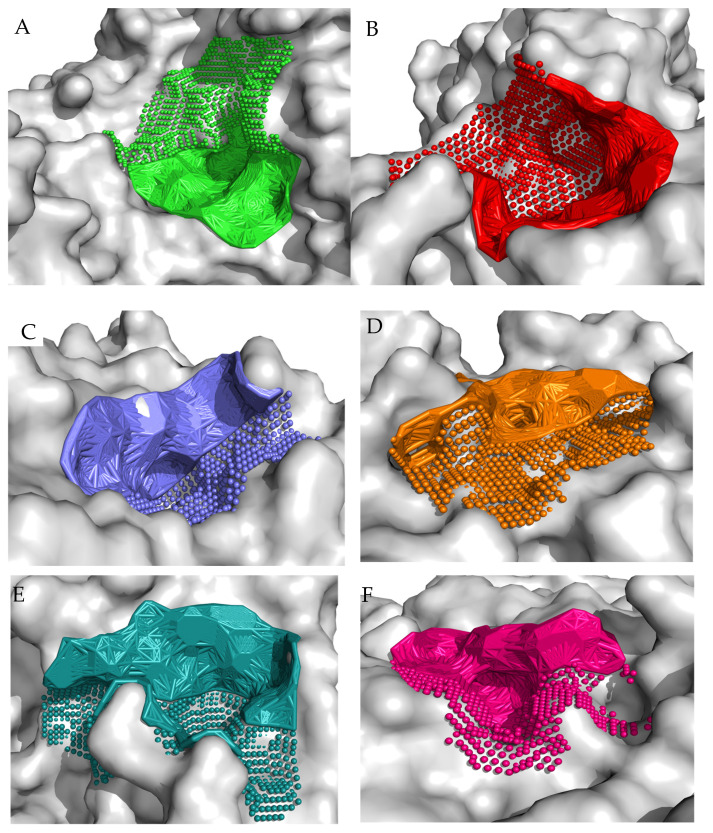

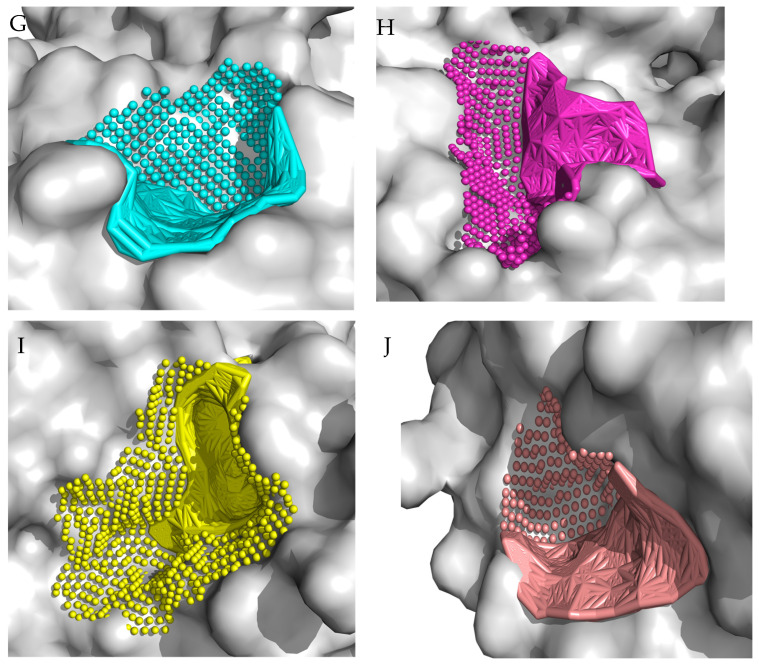
The best ten identified binding regions (**A**–**J**) on the molecular surface of the receptor. (**A**) The binding pocket is formed by the following amino acid residues: E346, P347, K348, S349, V350, Q352, Y360, H708, T711, L712, F713, H714, Q715, A716, V718, A719, G720, E721, T722, V723, R725, L729, E730, F731, P732, K733, W746, G747, E748, A749, L750, L769, G770, T771, L818, R819, A820, G821, Y822, I823, I824, P825, A846, L847, T848, G851, E852, A853, R854, G855, E856, L857, F858, L868, Y873, Q875, V876, I877, F878, L879, A880, Arg881, and V890; (**B**) The binding pocket is formed by the following amino acid residues: E196, Q352, Y354, L355, D356, V357, V358, G359, Y360, P361, F362, M363, P364, P365, I581, H584, R585, A586, L587, V588, K589, G592, T593, R594, P595, G607, R608, Y609, Y710, F713, H714, H717, V718, A719, G720, F858, D860, E863, S864, L865, E866, V867, L868, E869, R870, A872, and Y873; (**C**) The binding pocket is formed by the following amino acid residues: R281, D282, L283, A284, P285, Y292, W376, D404, L405, R411, I441, D443, K479, W481, W516, D518, M519, S523, N524, F525, I526, A554, A555, T556, R600, W613, G615, D616, V617, W618, D645, F649, L650, G651, N652, R672, H674, N675, S676, L677, L678, and S679; (**D**) The binding pocket is formed by the following amino acid residues: M146, Y148, R168, L169, D170, V171, M172, M173, E174, T175, R178, H180, F181, T182, I183, K184, R189, R190, Y191, E192, V193, P194, L195, E196, L246, L312, L313, N314, S315, N316, S332, G334, G335, I336, L337, D338, Y340, Q353, L355, D356, V357, V358, G359, Y360, N570, G605, R608, and Y609; (**E**) The binding pocket is formed by the following amino acid residues: P161, K162, D163, I164, L165, T166, K184, D185, A187, N188, R189, R190, Y191, E192, V193, P194, L195, F241, A242, D243, Q244, N316, T333, G334, G335, I336, T491, N536, E537, L538, E539, A559, S560, S561, H562, Q563, F564, L565, S566, T567, H568, Y569, N570, L571, and L574; (**F**) The binding pocket is formed by the following amino acid residues: G123, Q255, I257, T258, G259, L260, A261, E262, H263, L264, S265, P266, L267, M268, L269, S270, T271, S272, W273, T274, R275, I276, T277, L278, T286, P287, G288, A289, N290, L291, D319, V320, L322, P545, G546, V547, V548, E622, Q623, A625, S626, V628, P629, E630, I631, L632, Q633, F634, L637, T739, D741, and H742; (**G**) The binding pocket is formed by the following amino acid residues: W376, G377, Y378, S379, S380, D404, L405, D406, Y407, M408, D409, S410, R411, R412, F416, N417, K418, D419, G420, F421, W481, and L677; (**H**) The binding pocket is formed by the following amino acid residues: P266, W621, E622, A625, S626, S736, T737, W738, T739, V740, D741, H742, Gln743, Ile752, T753, P754, V755, L756, Q757, A758, K760, A761, E762, V763, T764, G765, Y766, W804, T806, L807, and A809; (**I**) The binding pocket is formed by the following amino acid residues: F128, F129, P130, P131, S132, Y133, P134, S135, R154, S214, E216, P217, F218, V230, N233, T234, T235, V236, A237, P238, L239, T250, S251, L252, P53, S254, Q255, Q323, P324, S325, P326, A327, L328, Q81, C82, D83, V84, P85, N87, S88, R89, and F90; (**J**) The binding pocket is formed by the following amino acid residues: R375, W376, G377, Y378, S379, A382, I383, T384, R385, Q386, V387, V388, N390, D406, N675, S676, L677, L678, S679, L680, P681, Q682, E683, Y685, S686, and F687.

Molecular docking is a very important approach used to better understand the binding mode between a ligand and a protein, thus determining the molecule that has the best interactions with the receptor. According to literature data, the binding pose for the 5NN8 receptor is situated in the following regions: Trp376, Tyr378, Leu405, Trp481, Asp518, Met519, Phe525, Asp616, Trp618, Phe649, Leu650, His674, and Leu678 [44]. In addition, we used three different types of computational software to predict the potential binding poses on the surface of the studied protein. The first software used was fpocket, which is an open-source package for detecting pockets in proteins. It is based on Voronoi tessellation and α spheres, built on top of the publicly available Qhull package. Given the structure of a protein, it enables the identification of potential binding sites. The fpocket analysis was performed using default settings, identifying 41 potential binding sites. The second program utilized was CAVITY software (version 1.0), which is specifically designed for the detection and analysis of ligand-binding sites. CAVITY is a geometry-based method that incorporates a spherical probing of the protein surface to detect potential binding sites. The CAVITY analysis was performed using the ‘whole protein detection mode’ and a ‘large’ option of the detection mode, which is used for large and complex cavity detection. CAVITY identified 35 potential binding sites. The open-source GHECOM software (version 1.0) was also used in this study, which is designed for finding multi-scale pockets on the protein surface using mathematically derived morphology. It is based on an algorithm for the simultaneous calculation of multiscale pockets, using several different sizes of spherical probes. Based on the literature data and obtained computational results, we identified the top ten binding sites (Figure 4). The molecular surface of the receptor, to which the peptide was docked, displays the top ten binding sites. The choice of the cavity was arbitrary and was dictated by its size, shape, and the size of the peptide molecule. Target binding/catalytic residues in both ligands and receptor-active sites that dynamically interact with each other are shown in the supplement (Figure S1).

**Figure 5:**
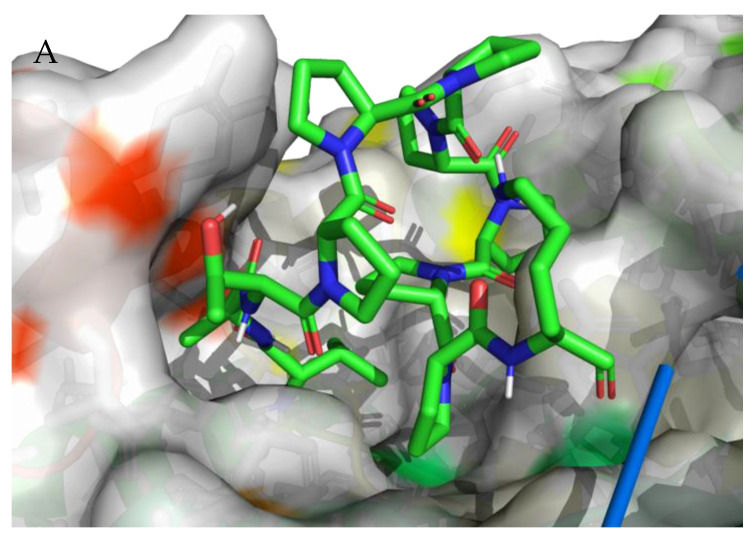

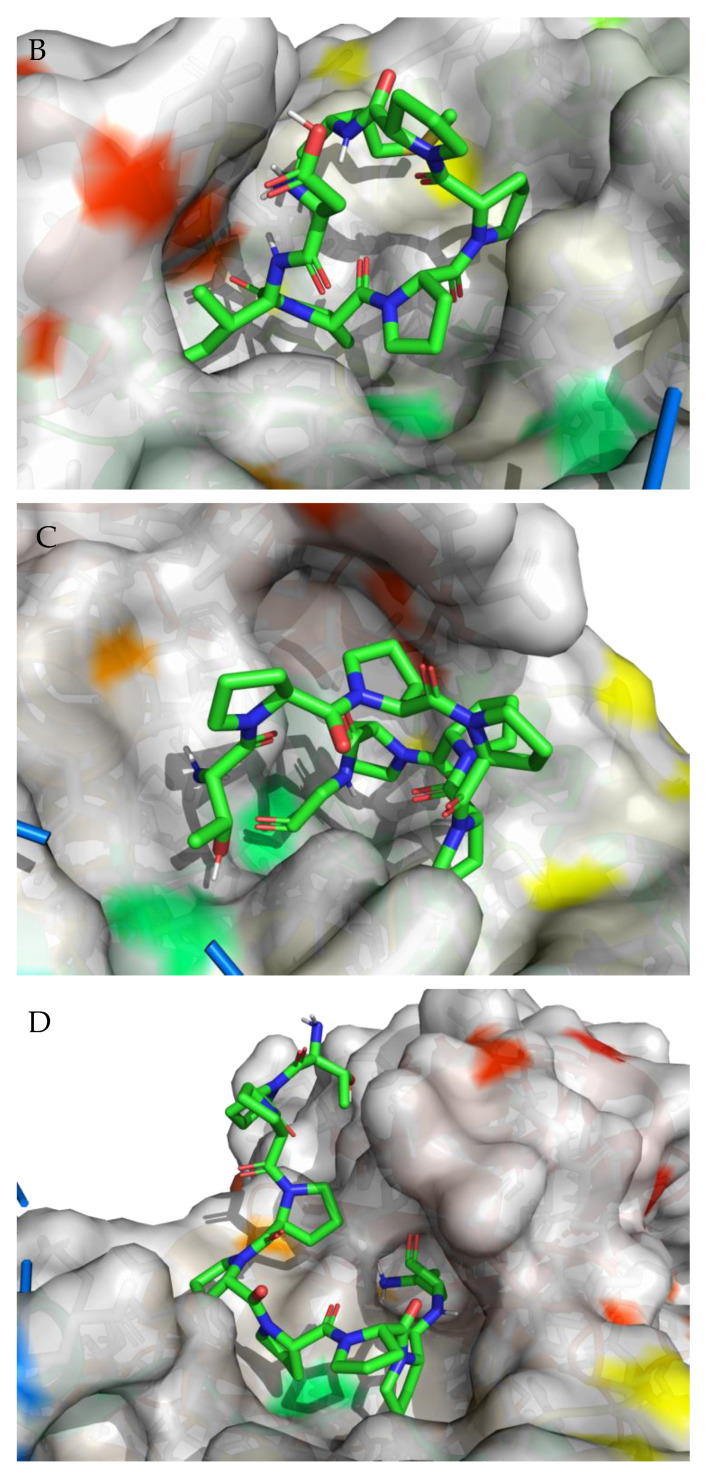
Projection of the best binding pocket of protein PDB ID: 5NN8 with docked ligands of sequence VATPPPPPPPK (**A**), DIPPPPM (**B**), TPPPPPPG (**C**), and TPPPPPPPK (**D**). In the peptide structure, the color green represents C atoms, blue represents N atoms, white represents H atoms, and red represents O atoms (Target binding/catalytic residues in both ligands and receptor active sites that dynamically interact with each other are shown in the supplement (Figure S2)).

For each of the peptides, the lowest ∆G binding energy was obtained for cavity pocket no. 2 (Table 3). This suggests that in this location, the peptides are most strongly attracted to the amino acid residues of the protein binding pocket. The projection of the best binding pocket of protein PDB ID: 5NN8 with docked ligands of sequence VATPPPPPPPK (A), DIPPPPM (B), TPPPPPPG (C), and TPPPPPPPK (D) is presented in Figure 5 with ∆G binding energy −6.6 kcal/mol, −6.7 kcal/mol, −7.0 kcal/mol, and −8.0 kcal/mol, respectively.

These results are close to those reported by Hu et al. [21]. The authors docked a promising molecular peptide from fermented rice bran, i.e., GLLGY, on human α-glucosidase (PDB ID 5NN8), and presented a binding energy of −7.1 kcal/mol. In addition, the authors of the study proved that this oligopeptide showed the greatest inhibitory activity in vitro, further highlighting the potential of the peptides presented in this study, obtained by hydrolysis of proteins from ripening raw loin, to bind to the human α-glucosidase molecule and act effectively as a potential antidiabetic agent.

The best binding site that was identified on the protein surface, with PDB ID: 5NN8, does not fully coincide with the literature data [44]. However, in the studies described in the cited source, protein 5NN8 interacted with a completely different ligand (i.e., the iridoid, Arbortristoside-C from Nyctanthes arbor-tristis Linn., which is a potential drug candidate for diabetes targeting α-glucosidase). Depending on the type of chemical compound, different binding pockets on the protein structure may be preferred. Furthermore, it should be noted that each computational program dedicated to molecular docking is based on different mathematical algorithms and scoring functions, which may lead to certain discrepancies in the resulting data.

## 4. Conclusions

The results indicate that peptides obtained by the hydrolysis of proteins for dry-cured pork loins may have potential as functional food ingredients in the prevention and/or treatment of type 2 diabetes mellitus. In particular, the docking studies on human α-glucosidase revealed that VATPPPPPPPK, DIPPPPM, TPPPPPPG, and TPPPPPPPK sequences are promising anti-diabetic candidates. These in vitro findings need further in vivo investigations to determine whether α-glucosidase inhibitory peptides could be used as agents for the prevention or treatment of type 2 diabetes.

## Figures and Tables

**Table 1:** List of peptide sequences (*A* parameter > 0.400) from dry-cured pork loins after hydrolysis.

No.	Peptides	*A* Parameter	MW [Da]	Protein ID	C_S	LOCK_S	BB_S	BAUER_S	C_M	LOCK_M	BB_M	BAUER_M
1	DIPPPPMDEK	0.400	1137.54	B5KJG2	+	+	+	+	−	−	+	−
2	EAPPPPAEVH	0.400	1042.51	Q75NG9	−	−	−	−	−	+	+	+
3	FDIPPPPMDE	0.400	1156.51	B5KJG2	−	−	−	+	−	−	−	−
4	SFDIPPPPMD	0.400	1114.50	B5KJG2	−	+	+	+	−	−	+	−
5	DLFPPPP	0.429	781.40	F1RYS7	−	−	−	+	−	−	−	−
6	IIAPPER	0.429	794.46	B6VNT8; C7AI81; F1SLG5; I3LVD5; P68137; Q6QAQ1	−	+	+	+	+	+	+	+
7	PPLIPPK	0.429	760.48	Q75ZZ6	−	−	−	−	+	−	−	−
8	FDIPPPPMD	0.444	1027.47	B5KJG2	−	−	−	+	−	−	−	−
9	IPPPPMDEK	0.444	1022.51	B5KJG2	−	−	−	+	−	−	−	−
10	RPPPISPPP	0.444	956.54	F1RNQ0; I3LH78; I3LL74	−	−	−	+	−	−	−	−
11	SFDIPPPPM	0.444	999.47	B5KJG2	−	+	−	−	−	−	−	−
12	KSLRSGLLGDTLTEGGLSQLGRALREL	0.476	2839.59	F1RGE5	+	−	−	−	−	−	−	−
13	VATPPPPPPPK	0.546	1096.63	I3LNG8	−	−	+	−	−	−	−	−
14	DIPPPPM	0.571	765.373	B5KJG2	−	−	−	+	−	−	−	−
15	TPPPPPPG	0.625	758.40	F1STN6	−	−	−	+	−	−	−	−
16	TPPPPPPPK	0.667	926.52	I3LNG8	−	−	−	+	−	−	−	−
				Total number	2	4	4	11	2	2	4	2

**Table 2:** Allergenicity and ADMET characteristics of selected peptide sequences with α-glucosidase inhibitor activity.

No. Peptides		Allergencity ^1^	A		D			M	E	T		
			Caco-2 Permeability ^2^	HIA ^3^	PPB ^4^	BBB ^5^	VD ^6^	Cyp450 2D6 ^7^ [I/S]	T_1/2_^8^	LD_50_^9^	H-HT ^10^	FDA ^11^
1	DIPPPPMDEK	Probable Non-Allergen	−6.522	0.270	52.70	0.076	−0.78	0.336/0.521	1.99	3.125	0.0	0.478
2	EAPPPPAEVH	Probable Allergen	−6.576	0.220	51.77	0.178	−0.74	0.376/0.473	1.92	3.226	0.0	0.31
3	FDIPPPPMDE	Probable Non-Allergen	−6.526	0.284	61.95	0.119	−0.87	0.407/0.488	1.95	3.157	0.0	0.346
4	SFDIPPPPMD	Probable Non-Allergen	−3.223	0.262	60.42	0.052	−0.85	0.375/0.479	1.93	3.178	0.0	0.308
5	DLFPPPP	Probable Non-Allergen	−3.185	0.208	62.08	0.256	−0.43	0.344/0.494	1.83	2.821	0.176	0.284
6	IIAPPER	Probable Allergen	−6.383	0.288	49.89	0.105	−0.52	0.359/0.526	1.68	2.838	0.056	0.418
7	PPLIPPK	Probable Non-Allergen	−5.963	0.312	56.01	0.079	−0.14	0.382/0.481	1.76	2.772	0.104	0.388
8	FDIPPPPMD	Probable Non-Allergen	−6.511	0.284	58.38	0.110	−0.80	0.384/0.499	1.88	3.143	0.0	0.324
9	IPPPPMDEK	Probable Non-Allergen	−3.292	0.270	50.21	0.094	−0.71	0.315/0.512	1.92	3.08	0.002	0.484
10	RPPPISPPP	Probable Non-Allergen	−6.631	0.280	51.28	0.024	−0.56	0.327/0.475	1.94	3.179	0.01	0.448
11	SFDIPPPPM	Probable Non-Allergen	−6.483	0.262	58.47	0.052	−0.76	0.387/0.478	1.90	3.102	0.0	0.306
12	KSLRSGLLGDTLTEGGLSQLGRALREL	Probable Allergen	−6.221	0.161	59.80	0.041	−0.25	0.445/0.437	2.15	3.239	0.0	0.44
13	VATPPPPPPPK	Probable Non-Allergen	−6.352	0.197	50.76	0.084	−0.30	0.343/0.518	2.08	3.314	0.0	0.428
14	DIPPPPM	Probable Non-Allergen	−6.122	0.278	47.39	0.175	−0.74	0.303/0.488	1.70	2.685	0.128	0.43
15	TPPPPPPG	Probable Non-Allergen	−6.132	0.165	43.80	0.548	−0.48	0.252/0.501	1.83	2.892	0.100	0.492
16	TPPPPPPPK	Probable Non-Allergen	−6.246	0.173	46.57	0.137	−0.34	0.270/0.548	1.99	3.218	0.024	0.472

^1^—allergenicity based on AllerTop 2.0; ^2^—Caco-2 Permeability [Expressed in cm × s^−1^], optimal: higher than −5.15 Log unit; ^3^—Human Intestinal Absorption, criteria: 0: HIA−(HIA < 30%), 1: HIA+ (HIA > 30%); ^4^—Plasma Protein Binding [%], optimal: <90%, significant with drugs that are highly protein-bound and have a low therapeutic index; ^5^—Blood–Brain Barrier (BBB), range: BB ratio ≥ 0.1: BBB+, BB ratio < 0.1: BBB−; ^6^—Value Distribution [L × kg^−1^], optimal: 0.04–20; ^7^—Cyp 450 inhibitor or substrate, criteria: 0: non-inhibitor/substrate, category 1: inhibitor/substrate; ^8^—Half Life, criteria: >8 h: high, from 3 h to 8 h: moderate, <3 h: low; ^9^—LD50 of acute toxicity [−log mol kg^−1^]; ^10^—Human Hepatotoxicity (H-HP), category 0: H-HT negative (−); Category 1: H-HT positive (+); ^11^—Maximum Recommended Daily Dose (FDAMDD), Category 0: FDAMDD negative (−); Category 1: FDAMDD positive (+).

**Table 3:** Free energy values for peptide binding to protein PDB ID 5NN8.

Cavity Number	∆G_binding_ [kcal/mol]			
	VATPPPPPPPK	DIPPPPM	TPPPPPPG	TPPPPPPPK
1	−4.7	−5.7	−6.7	−6.8
2	−6.6	−6.7	−7.0	−8.0
3	−4.2	−6.6	−6.9	−6.9
4	−4.3	−4.8	−5.7	−6.3
5	−5.8	−6.2	−6.7	−6.8
6	−5.3	−5.1	−6.7	−6.1
7	−2.2	−5.4	−5.5	−5.3
8	−4.8	−5.4	−5.8	−4.9
9	5.0	−5.8	−6.1	−5.5
10	36.9	−1.9	−1.4	−1.9

## Data Availability

All data are available in this study.

## Supplementary Materials

The following supporting information can be downloaded at: https://www.mdpi.com/article/10.3390/nu15163539/s1, Table S1: List of peptides with α-glucosidase inhibiting activity from dry-cured pork loins with strain of LAB.

## References

[1] Di Stefano E., Oliviero T., Udenigwe C.C. (2018). Functional significance and structure–activity relationship of food-derived α-glucosidase inhibitors. Curr. Opin. Food Sci..

[2] Ibrahim M.A., Bester M.J., Neitz A.W., Gaspar A.R. (2018). Structural properties of bioactive peptides with α-glucosidase inhibitory activity. Chem. Biol. Drug Des..

[3] Liu B., Kongstad K.T., Wiese S., Jäger A.K., Staerk D. (2016). Edible seaweed as future functional food: Identification of α-glucosidase inhibitors by combined use of high-resolution α-glucosidase inhibition profiling and HPLC–HRMS–SPE–NMR. Food Chem..

[4] Cisneros-Yupanqui M., Lante A., Mihaylova D., Krastanov A.I., Rizzi C. (2022). The α-Amylase and α-Glucosidase Inhibition Capacity of Grape Pomace: A Review. Food Bioproc. Tech..

[5] Kumar S., Narwal S., Kumar V., Prakash O. (2011). α-glucosidase inhibitors from plants: A natural approach to treat diabetes. Pharmacogn. Rev..

[6] Dirir A.M., Daou M., Yousef A.F., Yousef L.F. (2022). A review of alpha-glucosidase inhibitors from plants as potential candidates for the treatment of type-2 diabetes. Phytochem. Rev..

[7] Martínez-Sánchez S.M., Minguela A., Prieto-Merino D., Zafrilla-Rentero M.P., Abellán-Alemán J., Montoro-García S. (2017). The effect of regular intake of dry-cured ham rich in bioactive peptides on inflammation, platelet and monocyte activation markers in humans. Nutrients.

[8] Montoro-García S., Zafrilla-Rentero M.P., Celdrán-de Haro F.M., Piñero-de Armas J.J., Toldrá F., Tejada-Portero L., Abellan-Aleman J. (2017). Effects of dry-cured ham rich in bioactive peptides on cardiovascular health: A randomized controlled trial. J. Funct. Foods.

[9] Kęska P., Stadnik J. (2021). Potential DPP IV inhibitory peptides from dry-cured pork loins after hydrolysis: An in vitro and in silico study. Curr. Issues Mol. Biol..

[10] Kęska P., Stadnik J. (2022). Dipeptidyl Peptidase IV Inhibitory Peptides Generated in Dry-Cured Pork Loin during Aging and Gastrointestinal Digestion. Nutrients.

[11] Kęska P., Stadnik J., Bąk O., Borowski P. (2019). Meat proteins as dipeptidyl peptidase iv inhibitors and glucose uptake stimulating peptides for the management of a type 2 diabetes mellitus in silico study. Nutrients.

[12] Kęska P., Stadnik J. (2020). Structure–activity relationships study on biological activity of peptides as dipeptidyl peptidase IV inhibitors by chemometric modeling. Chem. Biol. Drug Des..

[13] Mora L., González-Rogel D., Heres A., Toldrá F. (2020). Iberian dry-cured ham as a potential source of α-glucosidase-inhibitory peptides. J. Funct. Foods.

[14] Molina I., Toldrá F. (1992). Detection of proteolytic activity in microorganisms isolated from dry-cured ham. J. Food Sci..

[15] Fadda S., Sanz Y., Vignolo G., Aristoy M.C., Oliver G., Toldrá F. (1999). Characterization of muscle sarcoplasmic and myofibrillar protein hydrolysis caused by Lactobacillus plantarum. Appl. Environ. Microbiol..

[16] Escudero E., Mora L., Toldrá F. (2014). Stability of ACE inhibitory ham peptides against heat treatment and in vitro digestion. Food Chem..

[17] UniProt KB. www.uniprot.org.

[18] BIOPEP-UWM. https://biochemia.uwm.edu.pl/biopep-uwm.

[19] ADMET. https://admet.scbdd.com.

[20] AllerTOP. https://www.ddg-pharmfac.net/AllerTOP/.

[21] Hu J., Lai X., Wu X., Wang H., Weng N., Lu J., Lyu M., Wang S. (2023). Isolation of a Novel Anti-Diabetic α-Glucosidase Oligo-Peptide Inhibitor from Fermented Rice Bran. Foods.

[22] Wairata J., Sukandar E.R., Fadlan A., Purnomo A.S., Taher M., Ersam T. (2021). Evaluation of the antioxidant, antidiabetic, and antiplasmodial activities of xanthones isolated from Garcinia forbesii and their in silico studies. Biomedicines.

[23] Roig-Zamboni V., Cobucci-Ponzano B., Iacono R., Ferrara M.C., Germany S., Bourne Y., Parenti G., Moracci M., Sulzenbacher G. (2017). Structure of human lysosomal acid α-glucosidase—A guide for the treatment of Pompe disease. Nat. Commun..

[24] Sanner M. (1999). Python: A Programming Language for Software Integration and Development. J. Mol. Graph. Model..

[25] Morris G., Huey R., Lindstrom W., Sanner M., Belew R., Goodsell D., Olson A. (2009). Autodock4 and AutoDockTools4: Automated docking with selective receptor flexibility. J. Comput. Chem..

[26] O’Boyle N.M., Banck M., James C.A., Morley C., Vandermeersch T., Hutchison G. (2011). Open Babel: An open chemical toolbox. J. Chem. Inform..

[27] Hassan N.M., Alhossary A.A., Mu Y., Kwoh C.-K. (2017). Protein-Ligand Blind Docking Using QuickVina-W With Inter-Process Spatio-Temporal Integration. Sci. Rep..

[28] Arnautova Y.A., Jagielska A., Scheraga H.A. (2006). A new force field (ECEPP-05) for peptides, proteins, and organic molecules. J. Phys. Chem. B.

[29] Le Guilloux V., Schmidtke P., Tuffery P. (2009). Fpocket: An open source platform for ligand pocket detection. BMC Bioinform..

[30] Zhang W., Yuan Y., Pei J., Lai L., Zhang W. (2015). CAVITY: Mapping the Druggable Binding Site. Computer-Aided Drug Discovery. Methods in Pharmacology and Toxicology.

[31] Kawabata T. (2010). Detection of multiscale pockets on protein surfaces using mathematical morphology. Proteins.

[32] Minkiewicz P., Iwaniak A., Darewicz M. (2019). BIOPEP-UWM database of bioactive peptides: Current opportunities. Int. J. Mol. Sci..

[33] Kęska P., Stadnik J. (2022). Peptidomic Characteristic of Peptides Generated in Dry-Cured Loins with Probiotic Strains of LAB during 360-Days Aging. Appl. Sci..

[34] Arámburo-Gálvez J.G., Arvizu-Flores A.A., Cárdenas-Torres F.I., Cabrera-Chávez F., Ramírez-Torres G.I., Flores-Mendoza L.K., Gastelum-Acosta P.E., Figueroa-Salcido O.G., Ontiveros N. (2022). Prediction of ACE-I inhibitory peptides derived from chickpea (*Cicer arietinum* L.): In silico assessments using simulated enzymatic hydrolysis, molecular docking and ADMET evaluation. Foods.

[35] Barrero J.A., Cabrera F., Cruz C.M. (2021). Gliptins vs. Milk-derived Dipeptidyl-Peptidase IV Inhibiting Biopeptides: Physicochemical Characterization and Pharmacokinetic Profiling. Vitae.

[36] Borawska-Dziadkiewicz J., Darewicz M., Tarczyńska A.S. (2021). Properties of peptides released from salmon and carp via simulated human-like gastrointestinal digestion described applying quantitative parameters. PLoS ONE.

[37] Kumar N., Goel N., Yadav T.C., Pruthi V. (2017). Quantum chemical, ADMET and molecular docking studies of ferulic acid amide derivatives with a novel anticancer drug target. Med. Chem. Res..

[38] Nisha C.M., Kumar A., Nair P., Gupta N., Silakari C., Tripathi T., Kumar A. (2016). Molecular docking and in silico ADMET study reveals acylguanidine 7a as a potential inhibitor of β-secretase. Adv. Bioinform..

[39] Altuner E.M. (2022). In Silico Proof of the Effect of Quercetin and Umbelliferone as Alpha-Amylase Inhibitors, Which Can Be Used in the Treatment of Diabetes. Kastamonu Univ. J. For..

[40] Guengerich F.P. (2008). Cytochrome p450 and chemical toxicology. Chem. Res. Toxicol..

[41] Mamadalieva N.Z., Youssef F.S., Hussain H., Zengin G., Mollica A., Al Musayeib N.M., Ashour M.L., Westermann B., Wessjohann L.A. (2021). Validation of the antioxidant and enzyme inhibitory potential of selected triterpenes using in vitro and in silico studies, and the evaluation of their ADMET properties. Molecules.

[42] Duchin K.L., McKinstry D.N., Cohen A.I., Migdalof B.H. (1988). Pharmacokinetics of captopril in healthy subjects and in patients with cardiovascular diseases. Clin. Pharmacokinet..

[43] Khaldan A., Bouamrane S., El Mchichi R.E.M.L., Maghat H., Lakhlifi M.B.T., Sbai A. (2022). In search of new potent α-glucosidase inhibitors: Molecular docking and ADMET prediction. Mor. J. Chem..

[44] Vajravijayan S., Nandhagopal N., Anantha Krishnan D., Gunasekaran K. (2022). Isolation and characterization of an iridoid, Arbortristoside-C from Nyctanthes arbor-tristis Linn., a potential drug candidate for diabetes targeting α-glucosidase. J. Biomol. Struct. Dyn..

