# Supplementary material for: Novel α-Glucosidase Inhibitory Peptides Identified In Silico from Dry-Cured Pork Loins with Probiotics through Peptidomic and Molecular Docking Analysis
Source: Nutrients. 2023 Aug 11;15(16):3539. doi: 10.3390/nu15163539 (PMC10460020; doi:10.3390/nu15163539)
Supplement: Supplementary file 1 [file nutrients-15-03539-s001.zip › Figure S1.pdf]

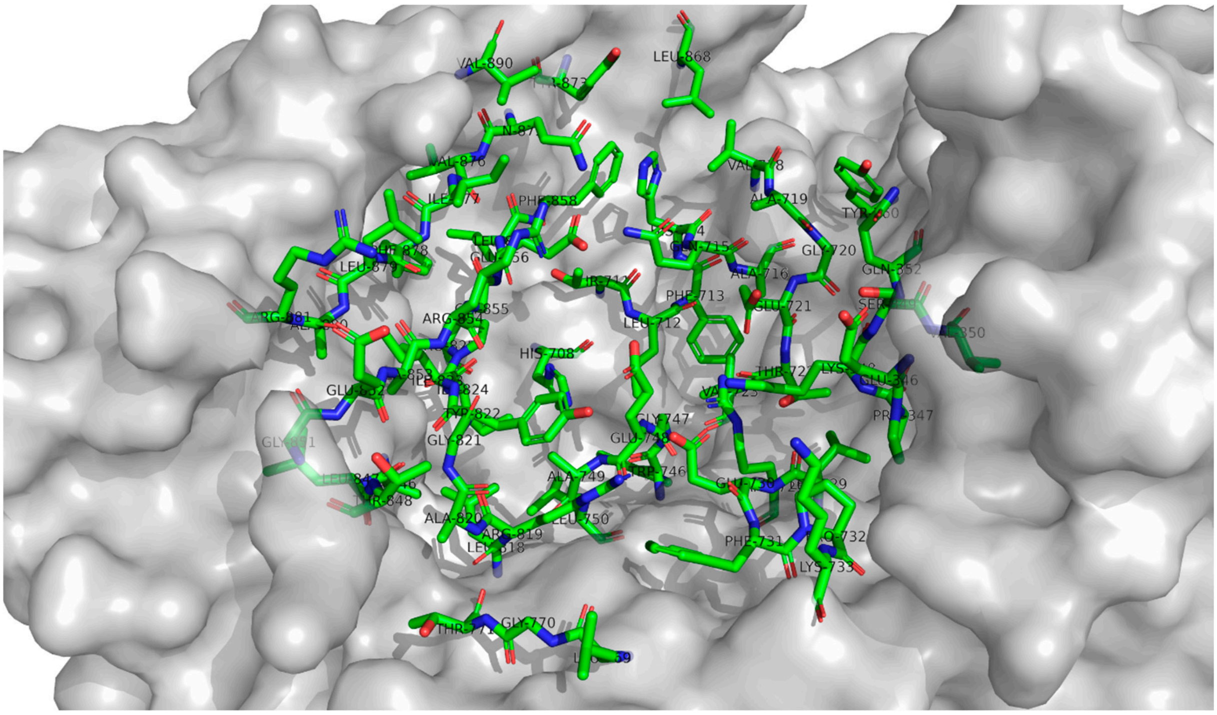

Figure S1_A. The binding pocket is formed by the following amino acid residues: E346, P347, K348, S349, V350, Q352, Y360, H708, T711, L712, F713, H714, Q715, A716, V718, A719, G720, E721, T722, V723, R725, L729, E730, F731, P732, K733, W746, G747, E748, A749, L750, L769, G770, T771, L818, R819, A820, G821, Y822, I823, I824, P825, A846, L847, T848, G851, E852, A853, R854, G855, E856, L857, F858, L868, Y873, Q875, V876, I877, F878, L879, A880, Arg881, V890.

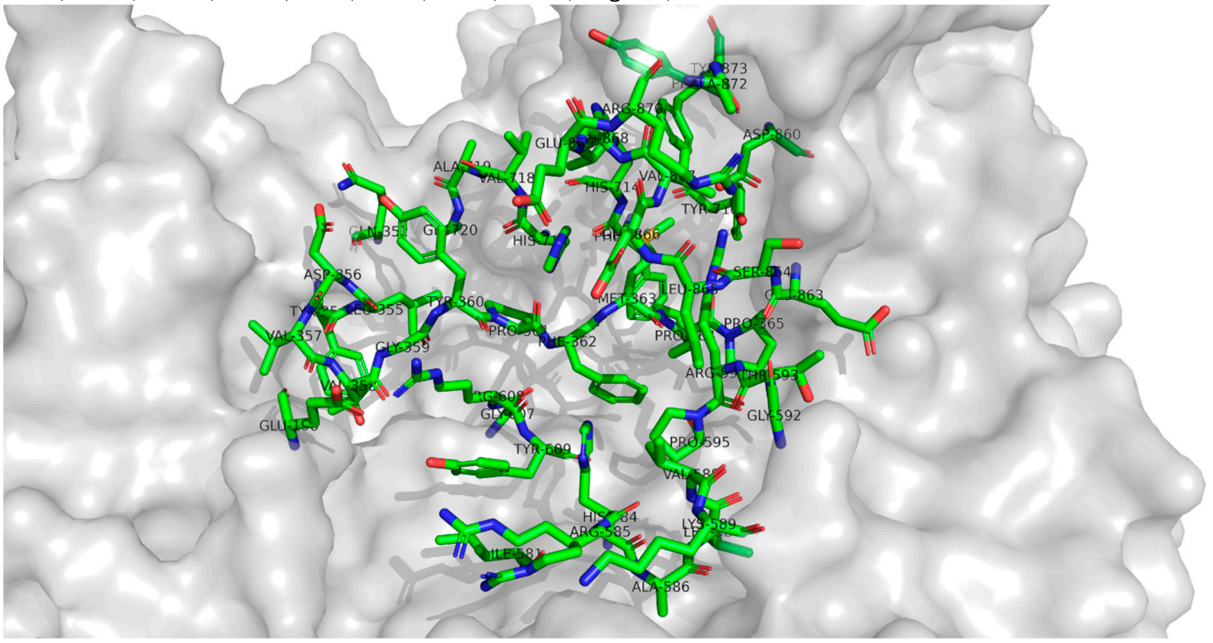

Figure S1_B. The binding pocket is formed by the following amino acid residues: E196, Q352, Y354, L355, D356, V357, V358, G359, Y360, P361, F362, M363, P364, P365, I581, H584, R585, A586, L587, V588, K589, G592, T593, R594, P595, G607, R608, Y609, Y710, F713, H714, H717, V718, A719, G720, F858, D860, E863, S864, L865, E866, V867, L868, E869, R870, A872, Y873.

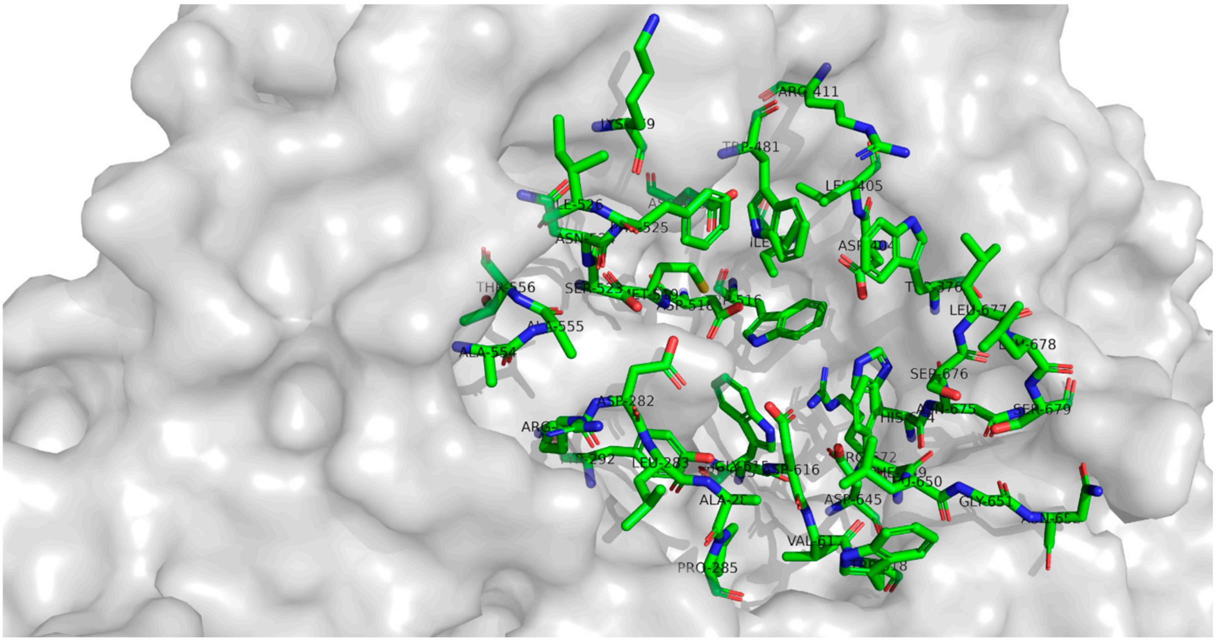

Figure S1_C. The binding pocket is formed by the following amino acid residues: R281, D282, L283, A284, P285, Y292, W376, D404, L405, R411, I441, D443, K479, W481, W516, D518, M519, S523, N524, F525, I526, A554, A555, T556, R600, W613, G615, D616, V617, W618, D645, F649, L650, G651, N652, R672, H674, N675, S676, L677, L678, S679.

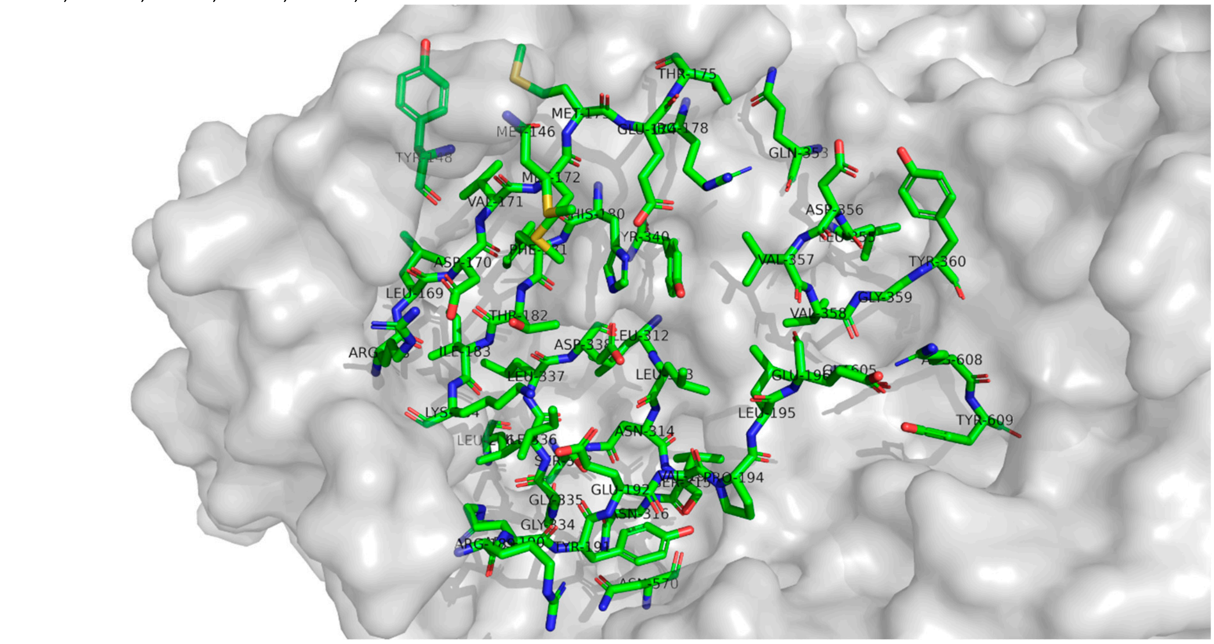

Figure S1_D. The binding pocket is formed by the following amino acid residues: M146, Y148, R168, L169, D170, V171, M172, M173, E174, T175, R178, H180, F181, T182, I183, K184, R189, R190, Y191, E192, V193, P194, L195, E196, L246, L312, L313, N314, S315, N316, S332, G334, G335, I336, L337, D338, Y340, Q353, L355, D356, V357, V358, G359, Y360, N570, G605, R608, Y609.

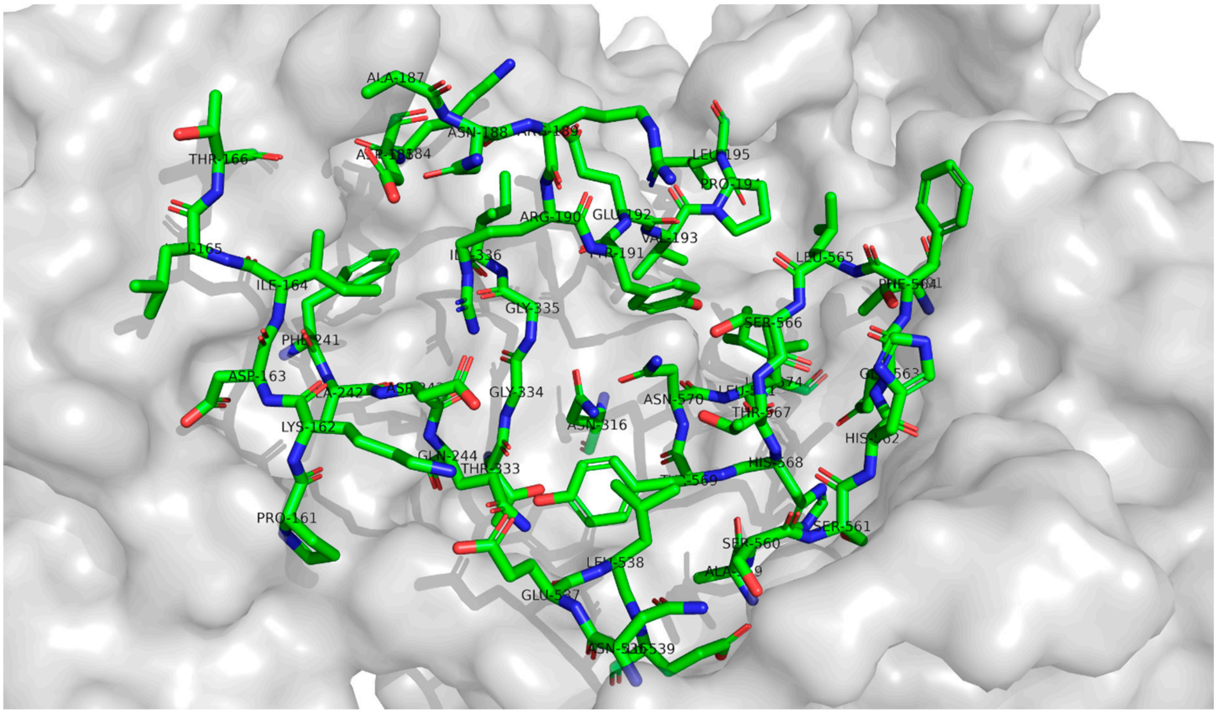

Figure S1_E. The binding pocket is formed by the following amino acid residues: P161, K162, D163, I164, L165, T166, K184, D185, A187, N188, R189, R190, Y191, E192, V193, P194, L195, F241, A242, D243, Q244, N316, T333, G334, G335, I336, T491, N536, E537, L538, E539, A559, S560, S561, H562, Q563, F564, L565, S566, T567, H568, Y569, N570, L571, L574.

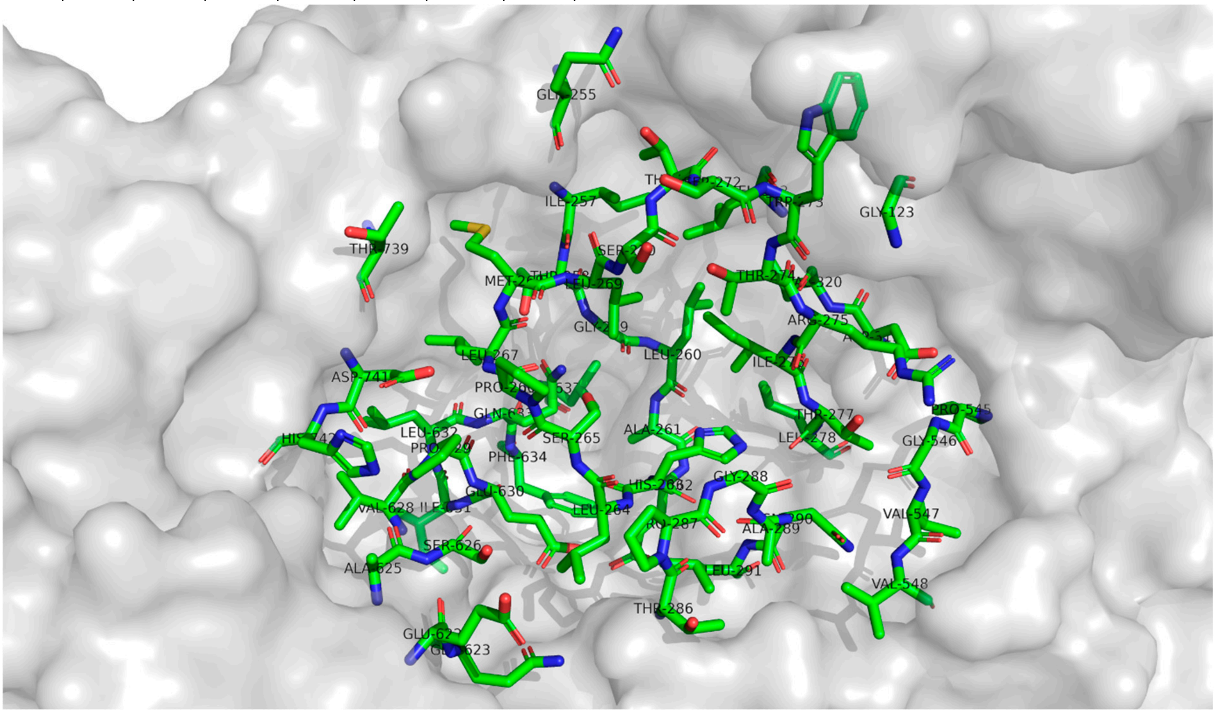

Figure S1_F. The binding pocket is formed by the following amino acid residues: G123, Q255, I257, T258, G259, L260, A261, E262, H263, L264, S265, P266, L267, M268, L269, S270, T271, S272, W273, T274, R275, I276, T277, L278, T286, P287, G288, A289, N290, L291, D319, V320, L322, P545, G546, V547, V548, E622, Q623, A625, S626, V628, P629, E630, I631, L632, Q633, F634, L637, T739, D741, H742

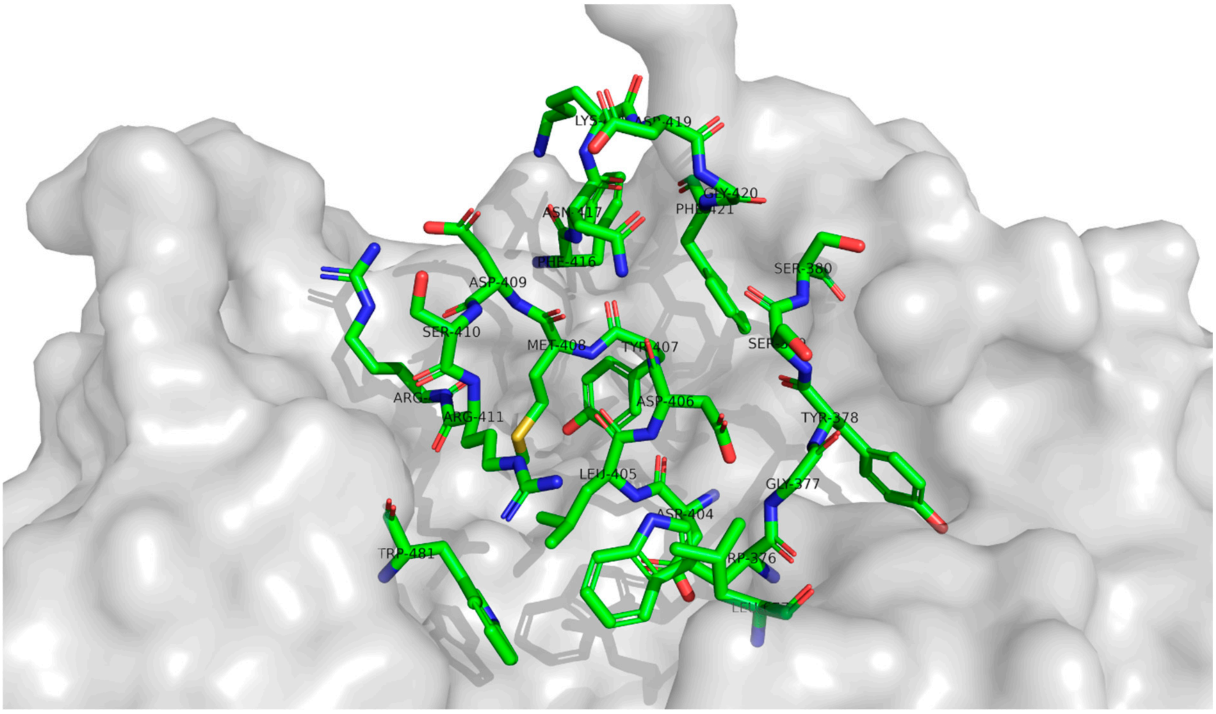

Figure S1_G. The binding pocket is formed by the following amino acid residues: W376, G377, Y378, S379, S380, D404, L405, D406, Y407, M408, D409, S410, R411, R412, F416, N417, K418, D419, G420, F421, W481, L677.

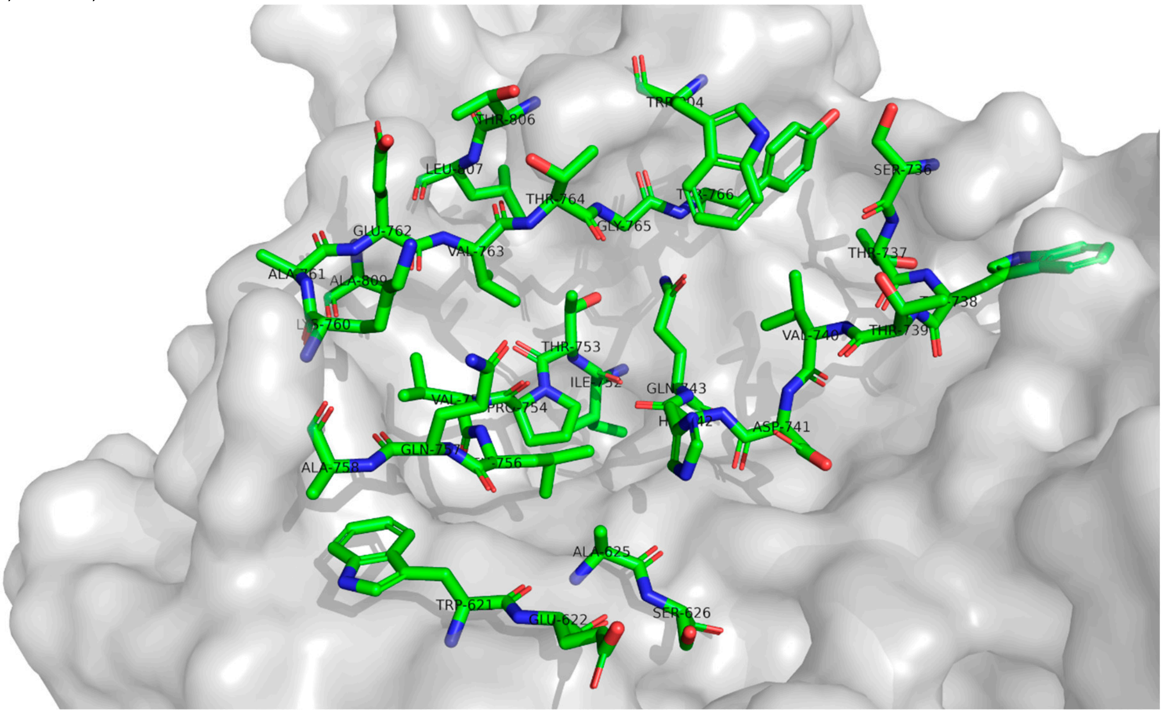

Figure S1_H. The binding pocket is formed by the following amino acid residues: P266, W621, E622, A625, S626, S736, T737, W738, T739, V740, D741, H742, Q743, I752, T753, P754, V755, L756, Q757, A758, K760, A761, E762, V763, T764, G765, Y766, W804, T806, L807, A809.

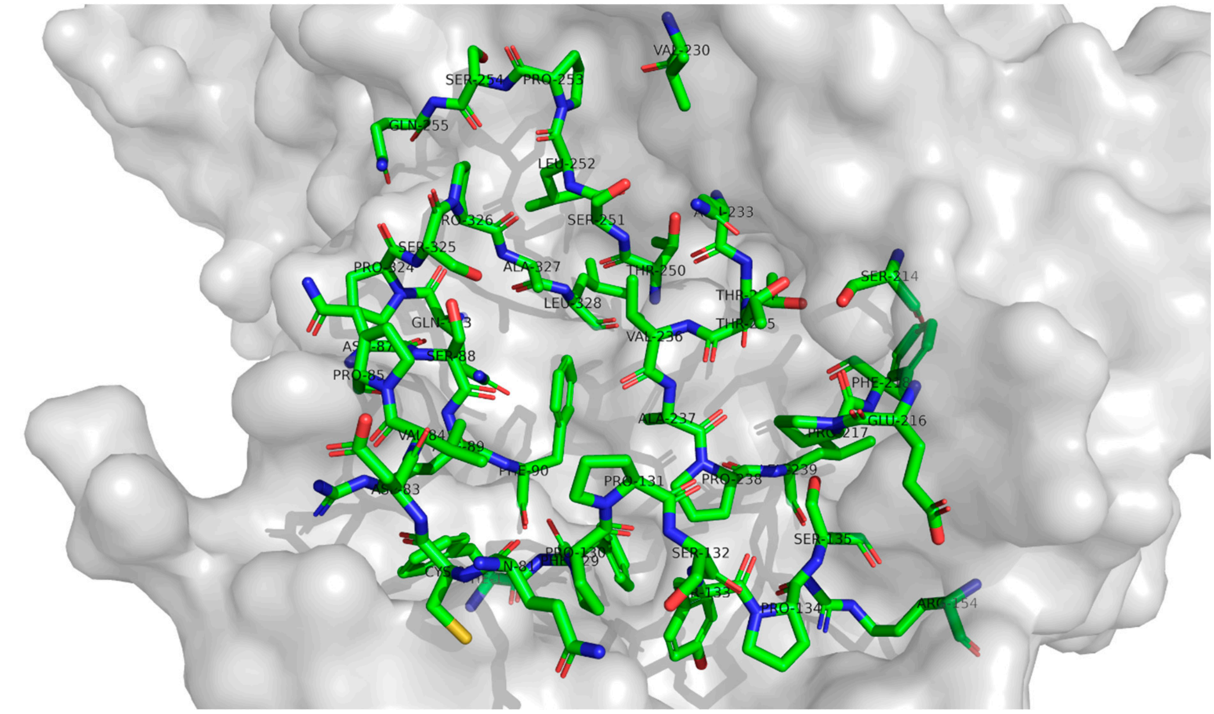

Figure S1_I. The binding pocket is formed by the following amino acid residues: F128, F129, P130, P131, S132, Y133, P134, S135, R154, S214, E216, P217, F218, V230, N233, T234, T235, V236, A237, P238, L239, T250, S251, L252, P53, S254, Q255, Q323, P324, S325, P326, A327, L328, Q81, C82, D83, V84, P85, N87, S88, R89, F90.

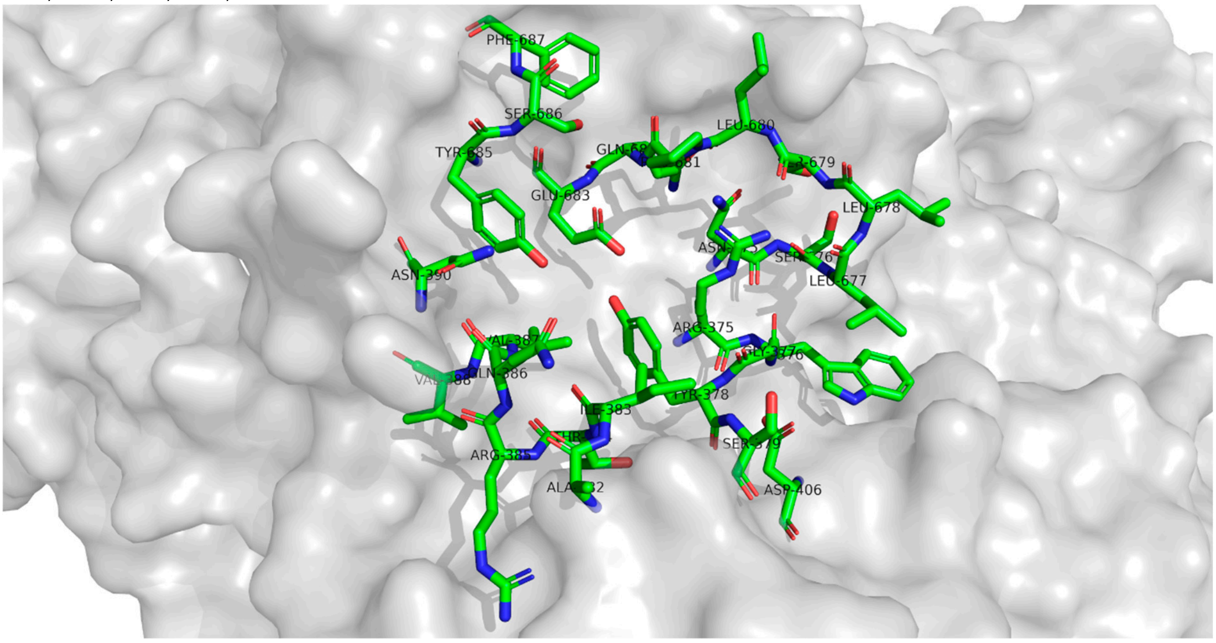

Figure S1_J. The binding pocket is formed by the following amino acid residues: R375, W376, G377, Y378, S379, A382, I383, T384, R385, Q386, V387, V388, N390, D406, N675, S676, L677, L678, S679, L680, P681, Q682, E683, Y685, S686, F687.
