# Supplementary material for: Novel α-Glucosidase Inhibitory Peptides Identified In Silico from Dry-Cured Pork Loins with Probiotics through Peptidomic and Molecular Docking Analysis
Source: Nutrients. 2023 Aug 11;15(16):3539. doi: 10.3390/nu15163539 (PMC10460020; doi:10.3390/nu15163539)
Supplement: Supplementary file 1 [file nutrients-15-03539-s001.zip › Figure S2.pdf]

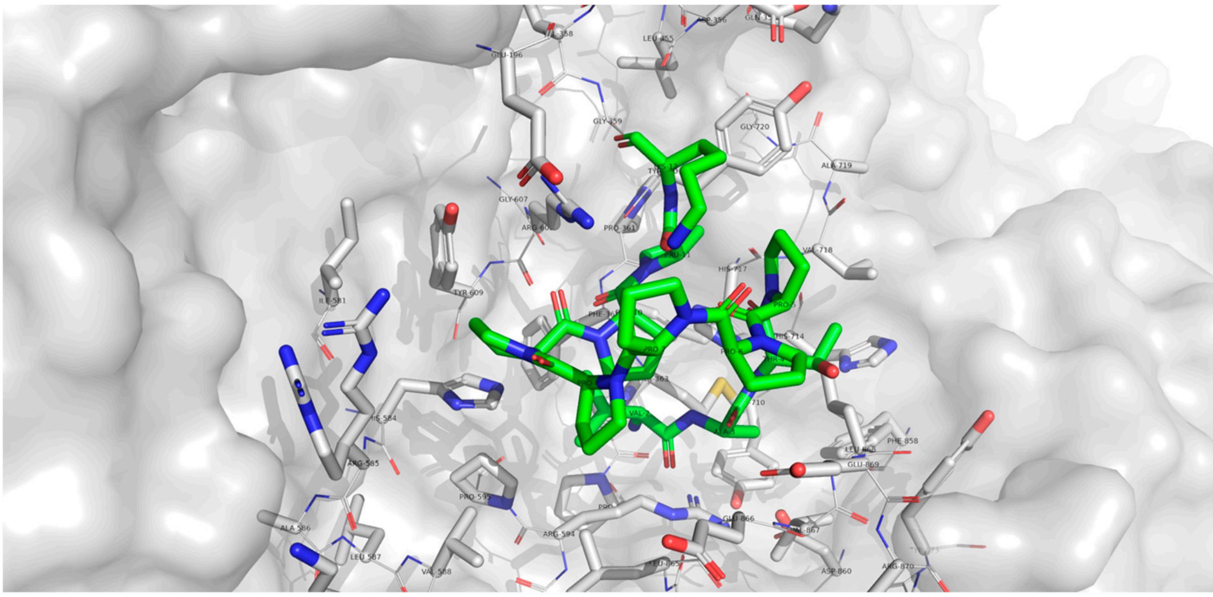

Figure S2_A. Projection of the best binding pocket of protein PDB ID: 5NN8 with docked ligand of sequence VATPPPPPPPPK. The structure of peptides is indicated by green color and gray color represents aminoacid residues, which building active pocket of protein. Hydrogen bonding interactions are between PRU11 (peptide) and ARG608 (protein), hydrophobic interactions are between PRU11 and Y360 (protein). Residue PRU is a modified proline, where the carbonyl CO group has been replaced by an OH group. Van der Waals interactions are between ALA3 (peptide) and LEU868 (protein). Electrostatic interactions are between LYS12 (peptide) and GLU196 (protein). Lack of disulfide bridges in active site of protein.

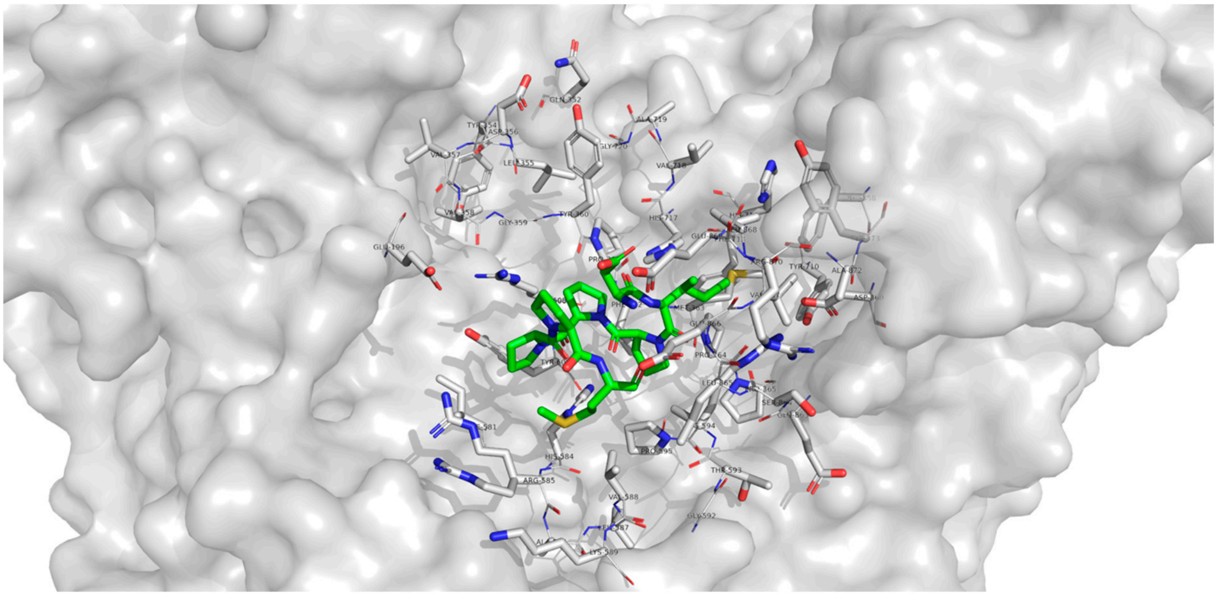

Figure S2_B. Projection of the best binding pocket of protein PDB ID: 5NN8 with docked ligand of sequence DIPPPPM. The structure of peptides is indicated by green color and gray color represents aminoacid residues, which building active pocket of protein. Lack of hydrogen bonds between the side chains of amino acid residues in the peptide and protein. Electrostatic interactions are between ASP2 (peptide) and GLU869 (protein). Van der Waals interactions are between MET8 (peptide) and VAL588 (protein), ILE3 (peptide) and LEU868 (protein). Lack of disulfide bridges in active site of protein.

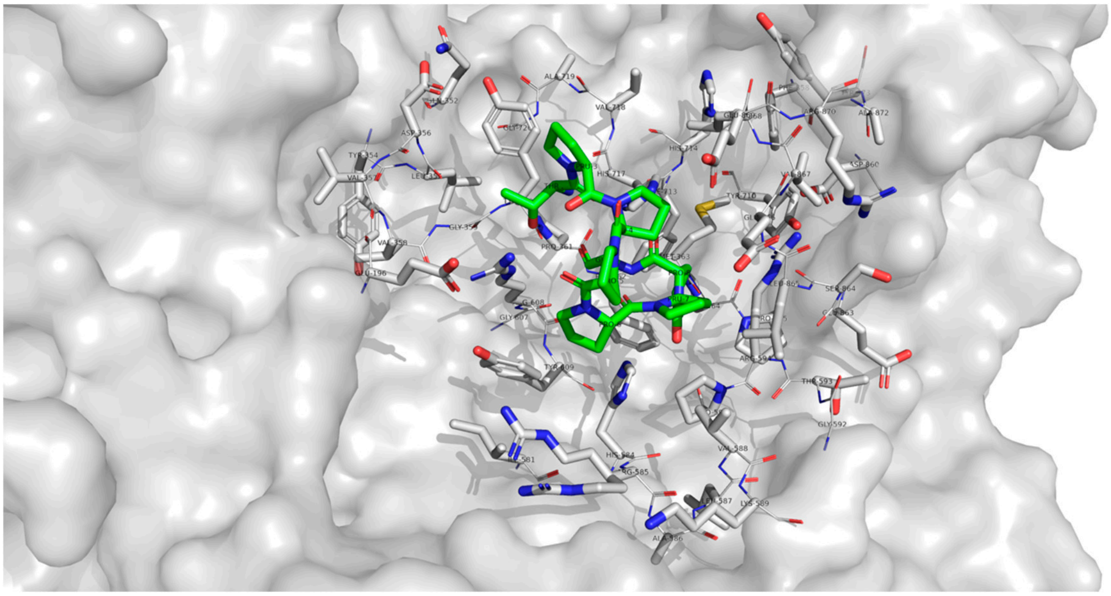

Figure S2_C. Projection of the best binding pocket of protein PDB ID: 5NN8 with docked ligand of sequence TPPPPPPG. The structure of peptides is indicated by green color and gray color represents aminoacid residues, which building active pocket of protein. Hydrogen bonding interactions are between PRU3 (peptide) and ARG608 (protein), hydrophobic interactions are between PRU11 (peptide) and GLU866 (protein) and PRU7 (peptide) and LEU865 (protein). Residue PRU is a modified proline, where the carbonyl CO group has been replaced by an OH group. Lack of disulfide bridges, and electrostatic interactions in active site of protein. Van der Waals interactions are between PRO8 (peptide) and MET363 (protein).

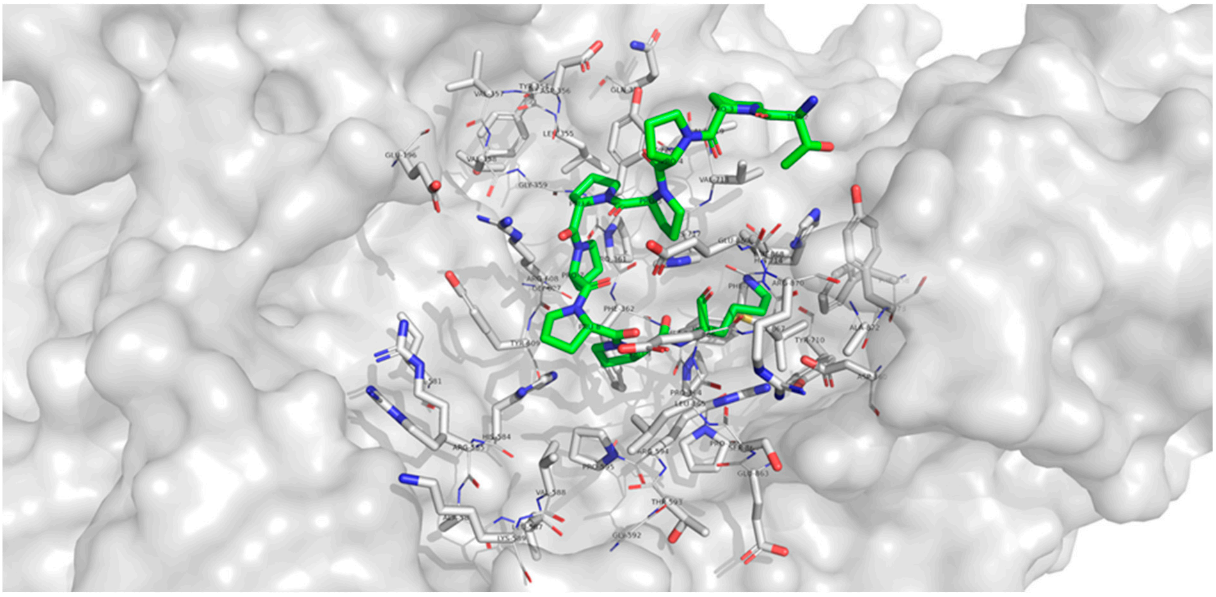

Figure S2_D. Projection of the best binding pocket of protein PDB ID: 5NN8 with docked ligand of sequence TPPPPPPPK. The structure of peptides is indicated by green color and gray color represents aminoacid residues, which building active pocket of protein. Hydrogen bonding interactions are between PRU6 (peptide) and ARG608 (protein), PRU8 (peptide) and GLU866 (protein), PRU9 (peptide) and MET363 (protein), hydrophobic interactions are between PRU6 (peptide) and TY360 (protein), PRU9 (peptide) and PHE362 (protein). Residue PRU is a modified proline, where the carbonyl CO group has been replaced by an OH group.

Electrostatic interactions are between LYS10 (peptide) and GLU866 (protein). Van der Waals interactions are between PRO3 (peptide) and VAL718 (protein, PRO5 (peptide) and LEU868 (protein). Lack of disulfide bridges in active site of protein.
